# SDACS: Blockchain-Based Secure and Dynamic Access Control Scheme for Internet of Things

**DOI:** 10.3390/s24072267

**Published:** 2024-04-02

**Authors:** Qinghua Gong, Jinnan Zhang, Zheng Wei, Xinmin Wang, Xia Zhang, Xin Yan, Yang Liu, Liming Dong

**Affiliations:** 1 State Key Laboratory of Information Photonics and Optical Communications, Beijing University of Posts and Telecommunications, Beijing 100876, China; qinghuagong@bupt.edu.cn (Q.G.); weizheng@bupt.edu.cn (Z.W.); wangxinmin@bupt.edu.cn (X.W.); xzhang@bupt.edu.cn (X.Z.); xyan@bupt.edu.cn (X.Y.); 2 School of Electronic Engineering, Beijing University of Posts and Telecommunications, Beijing 100876, China; 3 School of Automation, Beijing Institute of Technology, Beijing 100876, China; yangliu_npu@163.com; 4 Beijing Institute of Astronautical Systems Engineering, Beijing 100876, China; 5 Joint Logistics Academy of NDU, China People’s Liberation Army National Defence University, Beijing 100876, China; dlm14@tsinghua.org.cn

**Keywords:** attribute-based access control, blockchain, authentication, Internet of Things, smart contract, Hyperledger Fabric

## Abstract

With the rapid growth of the Internet of Things (IoT), massive terminal devices are connected to the network, generating a large amount of IoT data. The reliable sharing of IoT data is crucial for fields such as smart home and healthcare, as it promotes the intelligence of the IoT and provides faster problem solutions. Traditional data sharing schemes usually rely on a trusted centralized server to achieve each attempted access from users to data, which faces serious challenges of a single point of failure, low reliability, and an opaque access process in current IoT environments. To address these disadvantages, we propose a secure and dynamic access control scheme for the IoT, named SDACS, which enables data owners to achieve decentralized and fine-grained access control in an auditable and reliable way. For access control, attribute-based control (ABAC), Hyperledger Fabric, and interplanetary file system (IPFS) were used, with four kinds of access control contracts deployed on blockchain to coordinate and implement access policies. Additionally, a lightweight, certificateless authentication protocol was proposed to minimize the disclosure of identity information and ensure the double-layer protection of data through secure off-chain identity authentication and message transmission. The experimental and theoretical analysis demonstrated that our scheme can maintain high throughput while achieving high security and stability in IoT data security sharing scenarios.

## 1. Introduction

With the accelerated construction of network infrastructure, such as 5G and low-power wide-area networks, hundreds of the Internet of Things (IoT) terminal devices are connected to the network, thus generating tens of billions of IoT data [1]. The full utilization of IoT data can promote the cooperation between different devices, which is of great significance for promoting the intelligence of the IoT and providing faster problem solutions. However, due to the lack of a safe and trustworthy data sharing mechanism [2,3], the existing massive IoT data are exclusively analyzed and used internally by the data owners. The data lack circulation, and the synergy of the data cannot be fully utilized.

From the perspective of both data supply and demand sides, data sharing faces the following challenges: (1) The data owner has security and privacy concerns. The IoT data often contain a large amount of private information, such as identity information and address information. Achieving trustworthy data sharing in the IoT is a difficult problem due to the absence of trust between participants involved in data sharing. In addition, during the data transmission process, a malicious attacker is potentially able to perform a series of attacks, leading to user data loss and identity leakage. (2) Data users have concerns about utility and credibility. Traditional access control is usually performed by a central authority [4], and data are still managed by a semi-trusted intermediary, keeping the data sharing procedure unclear. When faced with an increasing number of false data and false recommendations, it is crucial to confirm the authenticity of data sources and the accuracy of data. (3) The shortcomings of the traditional IoT center architecture are exposed [5]. As the number of devices in the IoT grows exponentially, tasks such as data storage and processing are challenging for a single central server [6]. Once an error occurs in the central server, it is very likely to cause the entire network to collapse.

Technologies such as access control, encryption, and blockchain are fortunately capable of addressing the challenges faced by IoT data sharing. Access control stands out as a leading technology for securing IoT data, permitting users to access data within specified limits, legally [7,8,9]. It is widely recognized that conventional access control models provide only broad and coarse-grained access control, rendering them inadequate for the typical open environments of the IoT. Attribute-based access control (ABAC) realizes fine-grained and dynamic management of permissions by flexibly combining attributes, such as subjects, objects, permissions, and environments [10].

In order to enhance the security of ABAC, encryption technology and blockchain are introduced in the access control process. The distributed storage framework of the blockchain makes it difficult to lose on-chain data, ensuring the immutability and traceability of the data through the chain structure and hash algorithm. In addition, blockchain technology is able to solve the single point of failure problem caused by the application of centralized access control architecture. It should be noted that due to the open and transparent nature of blockchain, users storing and sharing data on the blockchain may lead to privacy leaks of stored data and user identities, which makes privacy protection particularly important. Therefore, the data to be shared can be encrypted and stored, and user identities can be managed in a trustworthy manner to achieve privacy protection.

In view of the above considerations, we propose a secure and dynamic access control scheme based on smart contracts, named SDACS. This scheme presents practical technical assistance for securely and efficiently sharing data among multiple parties within the IoT. Our contributions are listed as follows:

- SDACS integrates the interplanetary file system (IPFS), blockchain technology, and ABAC. The encrypted data are stored on the IPFS, effectively solving the problems of high data storage costs and data security. On the blockchain, the scheme enables the sharing of data storage addresses and data hash. The data access control process is automatically executed by blockchain, which solves the trust issues caused by third-party institutions.
- The access control scheme consists of APDC, DRMC, APMC, and DACC. The four smart contracts cooperate with each other to enable detailed and dynamic access control of data. For malicious data users, we design a punishment mechanism to impose corresponding behavioral restrictions through a misbehavior list. The data user can obtain correct data only through both on-chain authorization and off-chain identity authentication.
- We propose a lightweight, certificateless authentication protocol that enables bidirectional identity authentication between a trusted central authority and users. In order to ensure minimal leakage of user identity information, the true identity of the user is known only to himself and TCA. Our protocol not only ensures the legitimacy of the user’s identity source, but also verifies whether the data user has access rights to the data, which achieves double-layer protection of data.
- Our authentication scheme achieves unforgeability under adaptive selected message attacks. We develop a prototype system utilizing Hyperledger Fabric and Python. Theoretical analysis and experimental evaluation show that the performance of SDACS is effective for IoT data security sharing scenarios.

The subsequent sections of this paper are organized as follows. Section 2 describes the related work. Section 3 discusses the system architecture, basic algorithms, security model, and workflow of SDACS. In Section 4, we elaborate on the scheme. Section 5 presents the security analysis of SDACS. In Section 6, we evaluate the performance of the scheme based on the experiment results. Finally, in Section 7, we present a conclusion and discuss further work.

## 2. Related Work

Access control technology is an important means to ensure access and data security, and it has been widely used in various scenarios [11]. It restricts subjects’ access to objects through access control policies, thereby ensuring that the accessed data are used safely within the legal scope. However, current access control schemes face problems, such as single points of failure, low reliability, and difficult supervision. General solutions find it difficult to solve the above problems, and feasible methods, such as security certificates, are costly [12]. The characteristics of blockchain, such as decentralization, non-tampering, and traceability, allow these problems to be effectively solved. The access rights of data users can be enforced and controlled through smart contracts and consensus mechanisms. Therefore, many scholars have proposed schemes that combine the IoT and blockchain based on current methods of access control.

### 2.1. Blockchain-Based Access Control

Jiang et al. [13] proposed an attribute-based data access control scheme that simplifies the access management method. However, the entire access control process is performed off-chain, and the computing power of the blockchain is not fully utilized. Fugkeaw et al. [14] proposed a blockchain-based identity verification and access management system designed for Single Sign-On (SSO) access to data. In this system, access control for cloud resources by data users is managed through smart contracts, ensuring traceability throughout the access process. Moreover, the SSO authentication employs a hash-based token management to verify identities reliably. Despite these advancements, the storage of access control policies in cloud storage still poses a risk of data leakage. To ensure reliable access control for IoT data, Riabi et al. [15] integrated capability-based access control (CapBAC), identity-based access control (IBAC), and blockchain technology to facilitate trustworthy access management. Furthermore, they store topics and access permissions in an access control list (ACL), which data owners can incorporate into smart contracts. However, in existing large-scale IoT systems, creating an individual ACL for each user appears impractical due to the escalating storage costs on the blockchain as the number of user increases. In order to solve this problem, Sarfaraz et al. [16] developed an IoT access control model based on ABAC and blockchain. ABAC is used to restrict access to specific users with the necessary attributes in their certificates, eliminating the need to create ACLs or assign roles to all users. Zhang et al. [17] proposed an access control framework based on smart contracts. This framework is based on the Ethereum platform and realizes trusted access control through the cooperation of access control contracts, judgment contracts, and a registration contract. However, no work has been carried out to evaluate its feasibility.

Analysis: Current studies realize data access control by deploying access control policies in the blockchain, which solves the problems of privacy leakage and single point of failure of the traditional centralized IoT architecture. However, most schemes either have large storage overhead or build access control systems based on traditional blockchains, such as Ethereum. Each node of the traditional blockchain executes transactions sequentially, and system performance will not increase with the increase in the number of nodes, making it unsuitable for large-scale data access control scenarios.

### 2.2. Privacy Preservation in IoT Access Control

Secure data sharing in IoT is a major focus of ongoing research. In order to improve the security of access control systems, methods have been proposed to encrypt information or protect data in other ways. Li et al. [18] designed a dual-layer access control model based on attributes. The initial layer encrypts data by using attribute-based encryption algorithms, while the subsequent layer applies optimized smart contracts and coordination algorithms for access control, protecting the privacy of data sharing. Luo et al. [19] proposed a user data access control scheme based on smart contracts, using threshold secret sharing to optimize the data encryption and storage mechanism and reduce storage overhead. Hao et al. [20] designed a generic architecture for storing attribute-based resource access policies. Besides, the data owner assigns a set of attributes to the user through off-chain signatures, and data users can only gain access through both off-chain signatures and on-chain evaluations. Jambi et al. [21] proposed a lightweight and hierarchical access control method to safeguard the security of the IoT system. Their method utilizes edge blockchain managers for device authentication, manages ABAC policies through aggregated edge blockchain managers, and executes the access control process through a cloud federation blockchain manager. Ali et al. [22] presented an access control system that incorporates the use of ring signatures. This system enhances security and privacy through the application of smart contracts and encryption techniques. It employs ring signatures for the encryption and decryption of data, ensuring that only designated and authenticated users can access the signed data, while the identity of the signer remains concealed from the users. Nevertheless, it falls short in providing fine-grained access management.

Analysis: Current studies focus on protecting the data to be shared through encryption methods, while ignoring the privacy of transaction data in the blockchain. During the access control process, users inevitably need to provide identity data information to achieve data sharing, but there is a lack of specific research on how to minimize the disclosure of information to achieve privacy protection.

### 2.3. Secure Storage in IoT Access Control

During the process of data sharing, the data redundancy problem caused by distributed storage in the blockchain still needs to be solved. Liu et al. [23] proposed a blockchain-based access control scheme. To alleviate the storage burden on blockchain nodes, they opted to record the URL link of the data produced by devices on the blockchain. Moreover, their approach utilizes three smart contracts to handle requests from data users. However, the storage of data in the cloud introduces a heightened risk of data breaches. Similar to Liu’s scheme, Sun et al. [24] stored policy files in the local database, and the blockchain records the digest of policy files, access decisions for data users, and IoT entities’ attributes, which also needs to consider the security of data storage. IPFS is a decentralized storage protocol with the following advantages: (1) Each data block has a unique hash value as its identifier, and each node verifies whether the received data block is complete and correct based on the hash value, which effectively prevents the data from being tampered with or damaged during transmission. (2) Data are stored dispersedly on multiple nodes, and each node only needs to store part of the data it is responsible for, saving a lot of storage space [25]. Therefore, it is appropriate to apply IPFS to data storage in IoT scenarios. Zhao et al. [26] proposed an attribute-based access control scheme. Their approach stores encrypted data on IPFS, while hash values are stored on the blockchain. This storage method alleviates blockchain storage burdens and addresses the issue of single points of failure inherent in centralized data storage systems. Fan et al. [27] combined with the IPFS and blockchain technology to propose a secure storage and authorized access system for private information. The system uses the blockchain to save the hash value and access policy of encrypted private information, while the real private information is stored in IPFS.

Analysis: Most current studies store data in cloud servers or local databases. Centralized data storage is easily lead to single points of failure and data redundancy problems. IPFS solves these problems as a distributed storage platform for encrypted data. However, in existing research schemes based on IPFS and blockchain, the protection and privacy management of access policies have not received enough attention.

In summary, in the current access control research of IoT, blockchain guarantees the immutability of data. However, many studies fall short of addressing IoT needs because they ignore the bottleneck of large-scale data storage. In addition, the contradiction between the security and efficiency of data sharing in the access control process still needs to be resolved. A comparison of major blockchain-based access control schemes is shown in Table 1. Different from other studies, our proposed scheme can provide data privacy and security protection in large-scale data scenarios while achieving efficient access control. On the one hand, we designed an on-chain data access control scheme based on the ABAC model and high-performance Hyperledger Fabric, achieving dynamic and fine-grained access control through the mutual collaboration between four smart contracts. On the other hand, we set up a trusted third party to conduct off-chain security verification of the user’s identity data and access permissions based on a lightweight, certificateless authentication protocol, with the purpose of realizing the safe and reliable data sharing and privacy management while the user remains anonymous. Besides, for alleviating the storage pressure of the blockchain, we encrypted the data and stored it in IPFS. IPFS will fragment the file and copy multiple copies to its various nodes, reducing the possibility of data leakage.

## 3. System Model

### 3.1. System Architecture

**Figure 1:**
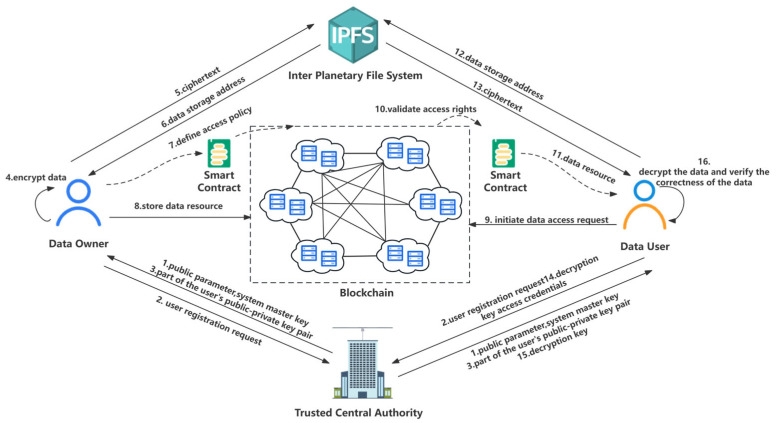
The architecture of SDACS.

Combined with the ABAC model in the IoT, we propose a data access control scheme based on blockchain, named SDACS. There are five main entities: trusted central authority, data owner, data user, blockchain, and IPFS. The detailed architecture of SDACS is shown in Figure 1.

- Trusted central authority (TCA): The TCA is a trusted organization, which is responsible for generating system parameters and performing user registration. Additionally, it provides identity authentication and decryption key generation services for the data user.
- Data owner (DO): The DO formulates the access policy for IoT data and encrypts the data based on parameters returned by the TCA. To improve storage efficiency, the DO sends the ciphertext to IPFS and synchronizes the data storage address and data hash, which we call data resource, to the blockchain through smart contracts. The DO is generally an IoT gateway device that connects device clusters to the blockchain network through wireless communication technology.
- Data user (DU): The DU sends a request to the DO to obtain data. The access behavior of the DU will be recorded in the blockchain, ensuring the traceability of the data resource access process. If the verification is successful, they will decrypt the ciphertext based on the decryption key sent by the TCA to obtain the data resource.
- Blockchain (BC): The BC is the core of the access control system. It is accountable for storing the storage address of data in the IPFS and data hash. As a fully trusted entity, all member nodes work honestly under the consensus mechanism. By deploying access control smart contracts, the blockchain system receives the DU’s access request, and automatically matches and verifies the attributes of the DU, which achieves fine-grained access control of the data.
- IPFS: The IPFS is a secure storage system that allows large-size IoT data to be stored for the DO. In addition, the DU can obtain ciphertext from the IPFS based on the storage address.

### 3.2. Basic Algorithms and Security Model

#### 3.2.1. Basic Algorithms

SDACS consists of seven basic algorithms.

$Setup(1^{\lambda })\to (pp,PK,MK)$: The TCA initialization algorithm inputs the security parameter λ, and outputs the public parameter pp, system master key PK, and system private key MK.

$Registration(pp,{ID}_{i},PK)\to ({UPK}_{i},{USK}_{i},\;{PID}_{i})$: The user registration algorithm inputs the public parameter pp, the user’s true identity ${ID}_{i}$, and the master key PK, and returns the public and private key pair $\left({UPK}_{i},{USK}_{i}\right)$ and the user’s pseudonym ${PID}_{i}$.

Encrypt(m,pp,PK)→(CT): The data encryption algorithm inputs data m, the public parameter pp, and the master key PK, and outputs the ciphertext CT.

$AuEncrypt(M,PK,pp,{PID}_{S},{USK}_{S},{PID}_{R},{UPK}_{R})\to (Cipher)$: The message authentication encryption algorithm takes the message M, the public parameter pp, the master key PK, the pseudonym ${PID}_{i}$ of the message sender and receiver, the sender’s private key ${USK}_{S}$, and the receiver’s public key ${UPK}_{R}$ as input parameters, and outputs the message ciphertext Cipher.

$AuDecrypt(Cipher,pp,PK,P{ID}_{S},{UPK}_{S},{PID}_{R},{USK}_{R})\to (M)$: The message authentication decryption algorithm inputs the message ciphertext Cipher, public parameter pp, master key PK, the pseudonym ${PID}_{i}$ of the message sender and receiver, the sender’s public key ${UPK}_{S}$, and the receiver’s private key ${USK}_{R}$, and outputs the message M.

$KeyGen(PK,MK,pp)\to ({SK}_{OwnerID})$: The decryption key generation algorithm inputs the master key PK, the system private key MK, and the public parameter pp, and returns the decryption key ${SK}_{OwnerID}$.

$Decrypt({SK}_{OwnerID},CT)\to (m)$: The ciphertext decryption algorithm inputs the decryption key ${SK}_{OwnerID}$ and ciphertext CT, and outputs the decrypted data m.

#### 3.2.2. Security Model

We utilized the security model proposed by Huang et al. [28] to assess the security of a lightweight, certificateless authentication protocol. In the security model, adversaries are divided into three security levels: normal, strong, and super adversary. If an authentication protocol is resistant to super adversaries, it means that it can also withstand attacks from powerful and normal adversaries.

We considered two types of adversaries, namely, adversary $A_{I}$ and adversary $A_{\mathrm{II}}$. $A_{I}$ can replace the public key, but it is not given TCA’s system private key. $A_{\mathrm{II}}$ has TCA’s system private key but cannot perform public key replacement. The specific security model is characterized by the game between challenger C and adversary $A_{I}$ or $A_{\mathrm{II}}$. The definition of the corresponding security game is provided below.

**Definition 1.**  *If there is no* $A_{I}$ *and* $A_{\mathrm{II}}$ *who can win two games with a non-negligible probability* ${Adv}_{A_{i}}(\lambda )$ *, where* i=1,2 *, we say that this lightweight, certificateless authentication protocol is existentially unforgeable against chosen-message attacks (EUF-CMA).*

Game I. The security game definition for adversary $A_{I}$ attack is as follows:

Initialization Phase. Challenger C runs the Setup() algorithm, inputs the security parameter λ, and returns the master key PK, public parameter pp, and system private key MK. Then, it sends PK and pp to $A_{I}$. MK will be kept secret.

Query Phase. $A_{I}$ performs queries of the following polynomial bounded number.

- Create-user query: $A_{I}$ inputs a query ID, and C searches the user list $L_{u}$ to confirm whether the user has been created. If so, C will return ${UPK}_{ID}$ to $A_{I}$. Otherwise, C will execute a series of algorithms to create users. Then, C adds $\left(ID,PID,d_{ID},x_{ID},{UPK}_{ID}\right)$ to $L_{u}$, and sends ${UPK}_{ID}$ and PID to $A_{I}$. It is assumed that create-user query is always asked before other oracles.
- Hash query: $A_{I}$ sends the relevant parameters to C to obtain the corresponding hash value.
- TCA partial private key query: $A_{I}$ requests the TCA partial private key of the user, whose pseudonym is PID. In response, C searches list $L_{u}$ and returns $d_{ID}$ to $A_{I}$.
- User private key generation query: $A_{I}$ requests the private key of the user, whose pseudonym is PID. The challenger C outputs the corresponding user private key x. If the public key is replaced and $A_{I}$ does not provide the corresponding $x^{\prime }$, then ⊥ is returned.
- User public key replacement query: For any user whose pseudonym is PID, the adversary $A_{I}$ can choose a new public key $\left(x^{\prime },{UPK}^{\prime }\right)$, and C will replace (x,UPK) of the user. In addition, $A_{I}$ may not provide $x^{\prime }$ corresponding to ${UPK}^{\prime }$. In this case, $x^{\prime }=⊥$ is returned.
- Message authentication encryption query: Challenger C runs the AuEncrypt() algorithm and outputs the message ciphertext, satisfying $Verify\left(PID,M,Cipher,pp,PK,{UPK}_{ID}\right)=1$, where ${UPK}_{ID}$ is the latest public key stored in $L_{u}$.

Forgery Phase. After the polynomial queries, for the message $M^{\prime }$, $A_{I}$ outputs a forged message ciphertext ${Cipher}^{*}$ for the user, whose pseudonym is PID. When the following three conditions are met at the same time, $A_{I}$ is said to win in Game I:

- $Verify\left(PID,M^{*},{Cipher}^{*},pp,PK,{UPK}_{ID}^{*}\right)=1$.
- $A_{I}$ has never submitted $\left(PID,M^{*}\;\right)$ to perform message authentication encryption queries.
- $A_{I}$ has never submitted PID to perform TCA partial private key queries.
  The probability of $A_{I}$ winning Game I is defined as ${Adv}_{A_{I}}(\lambda )$.

Game II. The security game definition for adversary $A_{\mathrm{II}}$ attack is as follows:

Initialization Phase. Challenger C runs the Setup() algorithm, inputs the security parameter λ, and returns the master key PK, public parameter pp, and system private key MK. Then, it sends PK, pp, and MK to $A_{\mathrm{II}}$.

Query Phase. $A_{\mathrm{II}}$ performs queries of the following polynomial bounded number.

- Create-user query: $A_{\mathrm{II}}$ takes as input a query ID, and C searches the user list $L_{u}$ to confirm whether the user has been created. If so, C will return ${UPK}_{ID}$ to $A_{\mathrm{II}}$. Otherwise, C will execute a series of algorithms to create users. Then, C adds $\left(ID,PID,\;d_{ID},x_{ID},{UPK}_{ID}\right)$ to $L_{u}$, and sends ${UPK}_{ID}$ and PID to $A_{\mathrm{II}}$. It is assumed that create-user query is always asked before other oracles.
- Hash query: $A_{\mathrm{II}}$ sends the relevant parameters to C to obtain the corresponding hash value.
- TCA partial private key query: $A_{\mathrm{II}}$ requests the TCA partial private key of the user, whose pseudonym is PID. In response, C searches list $L_{u}$ and returns $d_{ID}$ to $A_{\mathrm{II}}$.
- User private key generation query: $A_{\mathrm{II}}$ requests the private key of the user, whose pseudonym is PID. The challenger C outputs the corresponding user private key x. If the public key is replaced and $A_{\mathrm{II}}$ does not provide the corresponding $x^{\prime }$, then ⊥ is returned.
- User public key replacement query: For any user whose pseudonym is PID, the adversary $A_{\mathrm{II}}$ can choose a new public key $\left(x^{\prime },{UPK}^{\prime }\right)$, and C will replace (x,UPK) of the user. In addition, $A_{\mathrm{II}}$ may not provide $x^{\prime }$ corresponding to ${UPK}^{\prime }$. In this case, $x^{\prime }=⊥$ is returned.
- Message authentication encryption query: Challenger C runs the AuEncrypt() algorithm and outputs the message ciphertext, satisfying $Verify\left(PID,M,Cipher,pp,PK,{UPK}_{ID}\right)=1$, where ${UPK}_{ID}$ is the latest public key stored in $L_{u}$.

Forgery phase. After the polynomial queries, for the message $M^{\prime }$, $A_{\mathrm{II}}$ outputs a forged message ciphertext $\left(PID,M^{*},{Cipher}^{*}\right)$ for the user, whose pseudonym is PID. When the following three conditions are met at the same time, $A_{\mathrm{II}}$ is said to win in Game II:

- $Verify\left(PID,M^{*},{Cipher}^{*},pp,PK,{UPK}_{ID}^{*}\right)=1$.
- $A_{\mathrm{II}}$ has never submitted $M^{*}$ to perform message authentication encryption queries.
- $A_{\mathrm{II}}$ has never submitted PID to perform user private key generation queries.
- The probability of $A_{\mathrm{II}}$ winning Game II is defined as ${Adv}_{A_{\mathrm{II}}}(\lambda )$.

Unforgeability: If the success probability of any polynomial-time adversary is negligible in the above two games, then our lightweight, certificateless authentication protocol is unforgeable under adaptive chosen message attacks.

### 3.3. Workflow of SDACS

**Figure 2:**
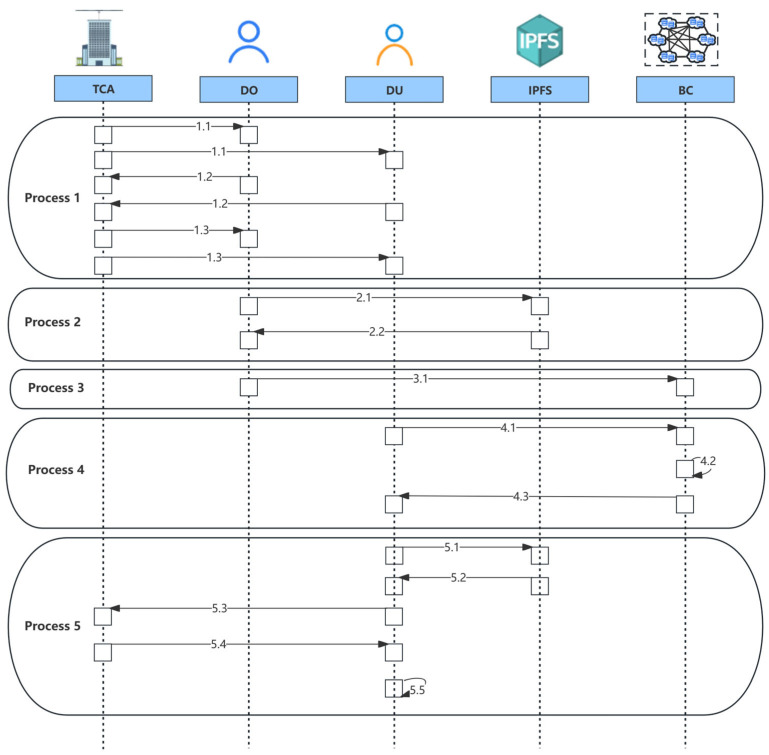
Workflow of SDACS.

The purpose of the SDACS is to build a safe, reliable, traceable, and confidential IoT data access control scheme. The scheme ensures the integrity of access data and implements fine-grained dynamic access control. Moreover, all access operations involved in the scheme are traceable and auditable. As is shown in Figure 2, the workflow of SDACS mainly contains five processes.

#### 3.3.1. Process 1: System Initialization and User Registration Process

The TCA generates public parameter pp, master key PK, and system private key MK, publishes PK and pp to the DU and DO, and retains the system private key MK.

The DO and DU register their identities in TCA.

The TCA generates the users’ public–private key pairs and sends them to the DO and DU. Then, the DO and DU generate their own public and private key pairs.

#### 3.3.2. Process 2: Data Encryption Process

The DO encrypts the data sent by IoT sensors and stores the ciphertext in IPFS. Besides, the DO generates data hash for subsequent authentication.

The IPFS sends the storage address Addr of the data to the DO.

#### 3.3.3. Process 3: Blockchain Initialization Process

The DO initiates the blockchain network and installs smart contracts that can enforce access control mechanisms for DU. After the invoke function is used to initialize the smart contracts, the DO can upload the data resource and access policy through AddData() and AddPolicy() methods. Here, the DO sets the user’s operation permissions for IoT data based on the ABAC model. Specifically, the DO extracts the attributes of subject, object, permission, and environment, respectively, transforms permission management into attribute management, thus forming an access policy. Besides, the DO is able to update and delete the access policy through the access policy management contract.

#### 3.3.4. Process 4: Access Control Process

The DU submits an access request to a specified data resource.

The access control contracts determine whether the DU has access rights to the data resource from four aspects: misbehavior verification, time verification, permission verification, and attribute matching.

If the permission verification is passed, the data resource is returned to the DU. However, if any of the links are invalid, the access verification process is stopped.

#### 3.3.5. Process 5: Data Decryption Process

The DU sends a data retrieval request to the IPFS based on the ciphertext storage address.

The IPFS returns ciphertext based on the ciphertext address.

The DU sends a decryption key request to the TCA. 

After the TCA ensures that the DU has obtained access rights by verifying the authenticity of the user’s identity, it sends the decryption key to the DU through a lightweight, certificateless authentication protocol.

The DU runs the decryption algorithm and obtains the data.

## 4. System Design

### 4.1. System Initialization

The system initialization algorithm is executed by the TCA. Specifically, the TCA releases the public parameter pp and master key PK with the given security parameter λ.

Firstly, the system selects the elliptic curve cyclic groups $G_{0}$ and $G_{1}$ with the order p of the security parameter λ; that is, $\left|p\right|=\lambda$. The generator of the group $G_{0}$ is P. $G_{0}$ and $G_{1}$ are the cyclic additive group and the cyclic multiplicative group, respectively, and satisfy bilinear mapping, $e{:G}_{0}\times G_{0}\to G_{1}$. Then, the TCA selects the hash function: $H_{1}:{\left\{0,1\right\}}^{*}\times G_{0}\times G_{0}\to Z_{p}^{*}$, $H_{2}:{\left\{0,1\right\}}^{*}\times G_{0}{\times \{0,1\}}^{*}\times G_{0}\times G_{0}\times G_{0}\to Z_{p}^{*}$, and $H_{3}:{\left\{0,1\right\}}^{*}\to {\left\{0,1\right\}}^{n}$, where n is the bit-length of the message. The TCA randomly selects $\alpha ,\beta ,s\in Z_{p}^{*}$ and outputs the public parameter $pp=\{G_{0},G_{1},P,e,p,H_{1},H_{2},H_{3}\}$, master key $PK=\{Q_{0}=e(P,P{)}^{\alpha },Q_{1}=\beta P,Q_{2}=sP\}$, and system private key MK={α,β,s}. Finally, the TCA publishes PK and pp, and retains MK.

### 4.2. User Registration

Generally speaking, the communication channels between off-chain institutions in the system are unreliable, and it is difficult to ensure the authenticity of a message during the transmission process. Without proper authentication mechanisms, the malicious adversaries are likely to intercept, modify messages being sent, and perform a range of attacks, which causes catastrophic damage to data owners and data users. Therefore, we propose a lightweight, certificateless authentication protocol to achieve secure two-way authentication between institutions.

We refer to the DO and DU collectively as users. It is assumed that the user registration phase takes place in a secure communication environment and data transmission is absolutely secure. In the identity authentication phase, attackers can eavesdrop, forge, or tamper with the transmitted messages, requiring institutions to authenticate and encrypt the communication information between each other. User registration is divided into the following steps:

User requests registration. The user submits a registration request to the TCA, which contains the real identification ${ID}_{i}$.

The TCA checks whether ${ID}_{i}$ has been used for registration. If not, the TCA generates pseudonym ($P{ID}_{i}$), for the users. It is assumed here that the pseudonym of the DO is OwnerID and the pseudonym of the DU is UserID. The purpose of this is to minimize the disclosure of information during the data sharing process and protect the privacy of user identities. Then, the TCA randomly selects $r_{i}\in Z_{p}^{*}$ and calculates $R_{i}=r_{i}P$, $h_{1}=H_{1}(P{ID}_{i}|\left|R_{i}\right||Q_{2})$, and $d_{i}=r_{i}+h_{1}s$. Finally, the TCA sends part of the user’s public–private key pair $\left(R_{i},d_{i},P{ID}_{i}\right)$ to the user.

User verification. The user calculates $h_{i}=H_{1}(P{ID}_{i}|\left|R_{i}\right||Q_{2})$ and verifies whether $d_{i}Q_{2}=R_{i}+h_{1}Q_{2}$ is true. If the equation holds, the protocol continues to generate the key. Otherwise, it returns ⊥ to indicate registration failure.

User key generation. After receiving $\left(R_{i},d_{i}\right)$, the user randomly selects $x_{i}\in Z_{p}^{*}$ and calculates $X_{i}=x_{i}P$. The user sets the user public key to ${UPK}_{i}=(R_{i},X_{i})$, the user private key to ${USK}_{i}=(d_{i},x_{i})$, and the pseudonym to $P{ID}_{i}$.

### 4.3. Data Encryption and Storage

The data encryption algorithm is executed by the DO. In order to prevent data leakage from the DO, after receiving the parameters sent by the TCA, the DO encrypts the data resource (m) using the public parameter pp and master key PK. The encryption process can be described as follows:

The DO randomly selects $\theta \in Z_{p}^{*}$, and calculates the ciphertext message $C_{m}$ and ciphertext parameters $C_{1},C_{2}$. The ciphertext output through the encryption algorithm can be expressed as: $CT=\{C_{m}=m\cdot e(P,P{)}^{\alpha \theta },C_{1}=-\theta \beta P,C_{2}=\theta P\}$. Besides, the DO executes $SHA256(DataID||{ID}_{DO})$ to generate the data hash (hashdata).

If the CT is stored directly in the blockchain, its size will inevitably put pressure on the node memory and reduce the performance of the blockchain. Therefore, we store CT in IPFS, and IPFS returns the corresponding storage address (Addr).

After completing the encryption and storage of data, the DO sets up the blockchain network. Since our scheme is based on Hyperledger Fabric to build a blockchain network, organizations joining the blockchain must have relevant certificates issued by a certificate authority (CA). The Hyperledger Fabric network we built includes two organizations, Org1 and Org2. Specifically, Org1 represents the DO, who owns data resources from IoT devices. Org2 represents the DU, who sends an access request to the data resource.

(1) CA→{Org1,Org2}

The DO formulates the access policy (Policy) for the data resource, specifies the access restriction conditions through Policy, and uploads it to the blockchain. Then, the DO stores the Addr and hashdata into the blockchain through the Data Resource Management Contract. Section 4.4 details the designed smart contracts.

### 4.4. Design of Smart Contracts

Our proposed access control scheme consists of four parts: the Access Policy Development Contract (APDC), Data Resource Management Contract (DRMC), Access Policy Management Contract (APMC), and Data Access Control Contract (DACC). Generally, the DO defines the access control policy of data through the APDC method. When the DU initiates a data resource access request, the DACC calls the access policy provided by the APMC based on the attribute set input by the DU to determine whether the DU has the permission to access the corresponding resource. If passed, the DACC retrieves the data resource from the DRMC, including the storage address of the data and data hash. The DU can obtain the ciphertext from the IPFS according to the data resource, and decrypt it to obtain the data.

(1) APDC: The APDC defines the access policy structure, DU’s attribute structure, data storage structure, and misbehavior record storage structure. Moreover, the APDC can only be executed by the DO to set the policy format. The access policy is usually represented by a set of attributes, which describes the information of data resource.

Policy={AS,AO,AP,AE}. The DO generates the key value, ResourceID=SHA256(OwnerID,DataID), associated with the access policy. The ResourceID and Policy can be expressed as ResourceID,Policy←{key:value}. The DU uses ResourceID as an identifier to associate with its own attribute set, and initiates data resource requests according to the defined request format.

As shown in Table 2, the DO divides the access policy model into Attribute of Subject (AS), Attribute of Object (AO), Attribute of Permission (AP), and Attribute of Environment (AE) in the APDC and defines the structure of each attribute.

AS includes the identity of the DO (OwnerID), owner department (Dep1), and owner role (Role1), and AO includes the identity of the data resource (DataID), the department to which the data belongs (Dep2), the role that generates data (Role2), and the address (Place). AP represents the data resource access permission, including allow (1) and deny (0). Besides, three parameters of AE are defined (i.e., CreationTime, EndTime, and Mode) to set the validity period of the access policy, with CreationTime and EndTime specified in unixtime format. Mode indicates whether the DU can dynamically access data resources, with 1 representing permission and 0 representing that the access policy is permanently valid.

${Req}_{ac}=\{{AS}_{u},{AO}_{u},UserID,Action\}$. The DU sends a request, ${Req}_{ac}$, to the blockchain. The GetUserReq() method extracts parameters such as ${AS}_{u}$, ${AO}_{u}$, and the identity of the DU (UserID) in ${Req}_{ac}$, then attaches a timestamp to record the request time. The blockchain sets the hash value of OwnerID and DataID as the ResourceID of the DU in the APDC according to the GetID() method, which is used for quick searching and adding the access policy.

Resource={Timestamp,DataResource}. In the blockchain network, the storage of data resources has been unanimously recognized by most nodes in the network. Therefore, the data resources stored in the blockchain can effectively prevent malicious tampering.

Record={UserID,Timestamp,DataID,Errortime}. When the DU sends access requests too frequently, it will be identified as a malicious user by the blockchain. At this time, the blockchain will automatically record the DU’s visit as an incorrect access behavior and store the inappropriate behavior through the AddMisbehavior() method, which mainly includes the UserID, DataID, the time when the misbehavior was exhibited (Timestamp), and the number of access errors committed by the malicious user (ErrorNumber).

(2) DRMC: The DRMC is used to manage the data resources. The DO can upload data resources through the AddData() method. When the blockchain system determines that the DU’s access is legal, the DACC returns the data resource to the DU by calling the GetData() method of the DRMC.

(3) APMC: The APMC is responsible for the policy management of the DO. Note that this smart contract converts the format of the access policy into a JavaScript object notation (JSON) object. As shown in Algorithm 1 and Algorithm 2, DO is able to implement the addition and deletion of the access policy through the AddPolicy() and DeletePolicy() methods respectively. Besides, the DO can quickly search and modify the formulated access policy through ResourceID in the QueryPolicy() method. If DO wants to update the policy, it can execute the UpdatePolicy() method. In the AddPolicy() method, the DO creates a new storage unit corresponding to the ResourceID and adds the access policy to the blockchain. If the ResourceID already exists, the access policy will be automatically updated.
**Algorithm 1** APMC.AddPolicy(): Add the policy into the blockchain network**Input:** Policy**Output:** Text or Error%policy=<AS, AO, AP, AE>ResourceID = SHA256 (Policy. AS. OwnerID + Policy. AO. DataID)**if** (QueryPolicy (ResourceID) == True) **then** % Policy already exists  UpdatePolicy (Policy)   **return** Text (“Policy is updated”)**else**  API.Putstate (ResourceID, Policy)   **return** Text (“Policy is added”)**endif**

**Algorithm 2** APMC.DeletePolicy(): Delete the policy from the blockchain network**Input:** Policy**Output:** Null or Error<AS, AO, AP, AE> ← PolicyResourceID = SHA256(Policy. AS. OwnerID + Policy. AO. DataID) APIStub.DelState(ResourceID, Policy)**return** null

(4) DACC: The core part of the smart contract system is the DACC. Algorithm 3 outlines the general process of the DACC processing an access request. The DACC performs access permission detection based on attribute matching through the AccessData() method, and maintains the misbehavior list of the DU at the same time. The request to access the data resource (${Req}_{ac}$) must contain exactly the same AS and AO attribute information listed in Table 2, plus the requested operation (Action). Here, ${Req}_{ac}$ can be expressed as $\left\{{AS}_{u},{AO}_{u},UserID,Action\right\}$.

**Figure 3:**
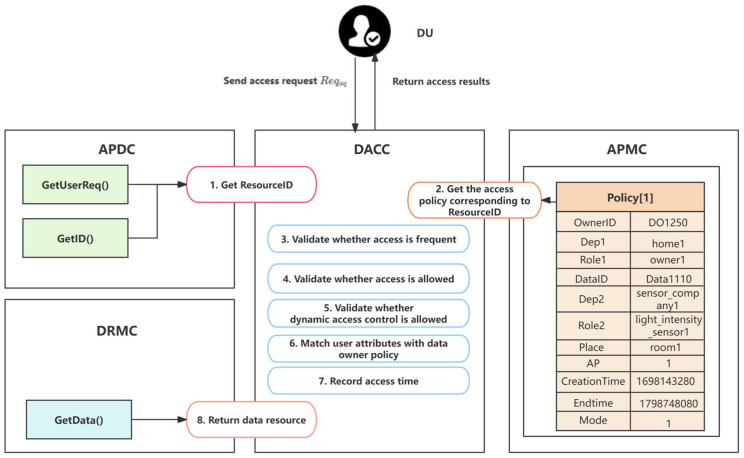
Access control process.

When the DACC receives the request ${Req}_{ac}$, it first calculates the ResourceID based on the DataID and OwnerID, then uses the ResourceID as input to call the APMC contract to retrieve the corresponding access policy. Afterward, the DACC determines whether the ${Req}_{ac}$ is allowed based on the rules in the policy. We assume that the DU with UserID “DU1001” requests to perform a read operation on the data resource with the DataID “4564784528”. We show the general procedure of DACC in Figure 3, and the detailed steps can be described as follows.

Step 1: The DACC calls the GetUserReq() and GetID() methods in the APDC to obtain ${AS}_{u},{AO}_{u}$, and UserID in the access request, and calculates the ResourceID based on the DataID and OwnerID.

Step 2: The DACC sends the ResourceID to the QueryPolicy() method of the APMC to query the related policy, ${policy}_{target}$. If there is no matching policy, the request’s handler should terminate.

Step 3: Based on the returned policy, the DACC determines whether the current access time of the DU is reasonably far from the last access time. When the DU is judged to be a frequent visitor, the SDACS system should restrict such behavior so that it cannot access the data resource within a period of time.
**Algorithm 3** DACC.AccessData(): Get Data Resource According to Policy.**Input:** Request of Data User**Output:** Data Resource or Error% MinTime represents the minimum allowed time interval for the data user, and the initial default value for UserID_LastTime is 0User_Policy = <${AS}_{u}$, ${AO}_{u}$, UserID> ← GetUserReq (User_Request) record ← GetMisbehavior(APIstub, UserID) **if** (record.ErrorTime < AllowedTimes) **then**
   **return** Text (“Access denied, access error limit reached”) **endif**Policy = <AS, AO, AP, AE> ← APIStub.InvokeChaincode (APMC.QueryPolicy, User_Policy.GetID())**if** (Time.Now()–UserID_LastTime < MinTime) then   AddMisbehavior(APIstub, UserID, Time.Now(), AO.DataID)  **return** Text (“Time interval is too short, please access again after a while”)**endif****if** (Policy. AP == 0) **then**  **return** Text (“Access Denied”) **endif****if** (Policy. AE. Mode == 1) **then**  **if** (Time.Now() > Policy. AE. EndTime) **then**   **return** Text (“Access Time Error”)  **endif**
**endif****if** ((User_Policy. ${AS}_{u}$ != Policy. AS) || (User_Policy. ${AO}_{u}$ != Policy. AO)) **then**  **return** Text (“Attribute Mismatch”)**endif**Data_Resource ← APIStub.InvokeChaincode (DRMC.GetData, User_Policy.AO. DataID) UserID_LastTime ← APIstub.PutState(UserID, Time.Now())**return** Data_Resource

In order to track a problematic DU, the DACC maintains a misbehavior list to save all inappropriate behaviors and return the results of the misbehavior to the DU, as shown in Table 3.

The DACC implements a misbehavior judgment method, and the punishment of the DU can be based on its wrong behavior history. Specifically, as shown in Algorithm 4, the DACC can record the DU’s inappropriate access behavior through the AddMisbehavior() method, and obtain the misbehavior history through the GetMisbehavior() method to determine the penalty for the DU.

Step 4: The DACC determines whether the data user is allowed to access the data resource based on the ${policy}_{target}$; that is, it checks whether the value of AP is 1, and otherwise, it will terminate the DU’s request program.

Step 5: If the value of the execution condition mode is 1, the DACC verifies whether the DU’s access time is within the time range specified in the access policy. If the access time is later than the policy revocation time, it means that the access policy has expired, and the requested process should be terminated.

Step 6: The DACC verifies whether the Dep1, Dep2, Role1, and Role2 entered by the DU are consistent with the existing policy. If any of the attributes matched result in false, the request program will be terminated.

Step 7: The DACC calls the DRMC to obtain the corresponding data resource based on the DataID, and sends it to the DU. Finally, the DU obtains the ciphertext from the IPFS based on the data resource and decrypts it by using the decryption key from the TCA.
**Algorithm 4** DACC. AddMisbehavior (): Add the misbehavior into the list of incorrect behaviors.**Input**: UserID, Timestamp, DataID**Output**: Null or Errorrecord = GetMisbehavior (UserID)Errortime = record.Errortime + 1Misbehavior = APDC. NewReocrd (UserID, Timestamp, DataID, Errortime) API.Putstate (UserID, Misbehavior)  **return** Null

### 4.5. Security Authentication and Key Generation

The DU submits the access credentials $Proof=\{OwnerID,DataID,UserID,hashdata,hashDU=SHA256({ID}_{DU})\}$ to the TCA to request the decryption key, ${SK}_{OwnerID}$, corresponding to the DataID. The TCA encrypts ${SK}_{OwnerID}$ based on Proof and sends it to the DU.

With the purpose of verifying the authenticity of the user’s identity and confirming that the DU has obtained access permission, the TCA first queries ${ID}_{DO}$ and ${ID}_{DU}$ based on OwnerID and UserID, and calculates and verifies whether $SHA256\left(DataID||{ID}_{DO}\right)=hashdata$ and $SHA256\left({ID}_{DU}\right)=hashDU$ are true. If established, the TCA executes the KeyGen() algorithm, inputs the master key PK, system private key MK, and public parameter pp, randomly selects $t\in Z_{p}$, calculates the private key parameters $D_{1}=\alpha P+\beta tP$ and $D_{2}=tP$, then outputs ${SK}_{OwnerID}=\{D_{1},D_{2}\}$.

To achieve safe message transmission, we need to authenticate the identities of both parties sending and receiving the message each time and encrypt the transmitted message to ensure its confidentiality. Since the steps of message encryption and decryption in the identity authentication process are the same, for the convenience of description, we simplified the identity authentication process between the DU and TCA to the mutual authentication between user Data_Sender and user Data_Receiver. Assuming that the identity of the message sender is ${PID}_{S}$, then the user’s public key is ${UPK}_{S}=\{R_{S},X_{S}\}$, and the user’s private key is ${USK}_{S}=\{d_{S},x_{S}\}$. Similarly, we suppose the identity of the message recipient is ${PID}_{R}$, then the user’s public key is ${UPK}_{R}=\{R_{R},X_{R}\}$, the user’s private key is ${USK}_{R}=\{d_{R},x_{R}\}$, and ${PID}_{S},P{ID}_{R}\in {\left\{0,1\right\}}^{*}$.

When Data_Sender sends a message M to Data_Receiver, it performs the following process based on the ${PID}_{R}$, ${UPK}_{R}$, and ${USK}_{S}$:

Data_Sender selects a random value $k\in Z_{p}^{*}$ and calculates T=kP.

Compute the shared secret value $V_{S}=k(X_{R}+R_{R}+h_{1}Q_{2})$, where $h_{1}=H_{1}(P{ID}_{R}|\left|R_{R}\right||Q_{2})$.

Compute $h_{2}=H_{2}(M|\left|T\right||P{ID}_{S}|\left|Q_{2}\right||R_{S}||X_{S})$ and $\delta =\frac{x_{S}+k+h_{2}d_{S}}{x_{S}}$.

Encrypt the message $C=H_{3}(V_{S})⨁(M)$, and send the message ciphertext Cipher=(T,C,δ) to the TCA.

When Data_Receiver receives the Cipher sent by Data_Sender, it performs the following process based on the $P{ID}_{S}$, ${UPK}_{S}$, and ${USK}_{R}$:

Compute the shared secret value $V_{R}=(x_{R}+d_{R})T$ and the recovery message $M=C⨁H_{3}(V_{R})$.

Compute $h_{1}^{\prime }=H_{1}(P{ID}_{S}|\left|R_{S}\right||Q_{2})$ and $h_{2}=H_{2}(M|\left|T\right||{PID}_{S}|\left|Q_{2}\right||R_{S}||X_{S})$, then verify $\delta X_{S}=X_{S}+T+h_{2}(R_{S}+h_{1}^{\prime }Q_{2})$. If the above equation holds, Data_Receiver receives M.

In our proposed protocol, it is assumed that the TCA is completely trustworthy. Moreover, the application of the protocol can be extended to any IoT scenario, even if the TCA is untrustworthy. In the case where neither of the identity authentication parties is a TCA, this protocol can simultaneously resist attacks by malicious message encryptors and malicious key generation centers. On the one hand, the message encryptor needs the TCA partial private key and the user key to generate valid message ciphertext. That is to say, an adversary who only possesses part of the TCA private key or the user’s private key cannot forge valid message ciphertext. On the other hand, to verify the validity of the message ciphertext, the master key, public parameters, and user public key need to be input simultaneously in the message authentication decryption algorithm, which means that the user private key and the TCA partial private key are properly combined in the generation of the message ciphertext. Therefore, our protocol is safe, trusted, and attack-resistant.

### 4.6. Data Decryption

The DU executes the decryption algorithm and inputs the ${SK}_{OwnerID}$ and CT, then the data is recovered, as computed:

(2) $$\begin{array}{ll} \frac{C_{m}}{e\left(C_{1},D_{2}\right)e\left(C_{2},D_{1}\right)} & =\frac{m\cdot e(P,P{)}^{\alpha \theta }}{e\left(-\theta \beta P,tP\right)e\left(\theta P,\alpha P+\beta tP\right)}\\ & =\frac{m\cdot e(P,P{)}^{\alpha \theta }}{{e\left(P,P\right)}^{-\theta \beta t}{e\left(P,P\right)}^{\alpha \theta }{e\left(P,P\right)}^{\beta \theta t}}=m \end{array}$$

Therefore, the DU completes the request for obtaining the required data.

## 5. Security Analysis

### 5.1. Security Analysis of Lightweight, Certificateless Authentication Protocol

#### 5.1.1. Proof of Correctness

(1) Correctness of the message ciphertext

In the AuEncrypt() algorithm, the verification of the message ciphertext can be completed by calculating the shared secret value, as shown in Equation (3):

(3) $$V_{S}=k\cdot \left(X_{R}+R_{R}+h_{1}Q_{2}\right)=k\cdot x_{R}P+k\cdot r_{R}P+k\cdot h_{1}sP=\left(x_{R}+d_{R}\right)T=V_{R}$$

(2) Correctness of message decryption

In the AuDecrypt() algorithm, the correctness of the decrypted message can be verified by Equation (4):

(4) $$\begin{array}{ll} \delta X_{S} & =\frac{x_{S}+k+h_{2}d_{S}}{x_{S}}\cdot x_{S}P\\ & =\left[x_{S}+k+H_{2}\left(m\left|\left|T\right|\right|{ID}_{S}\left|\left|Q_{2}\right|\right|R_{S}||X_{S}\right)\left(r_{S}+H_{1}\left({ID}_{S}\left|\left|R_{S}\right|\right|Q_{2}\right)s\right)\right]P\\ & =X_{S}+T+h_{2}\left(R_{S}+h_{1}^{\prime }Q_{2}\right) \end{array}$$

#### 5.1.2. Proof of Security

**Theorem 1.**  In the random oracle model, if the elliptic curve discrete logarithm problem (ECDLP) is difficult, then the lightweight, certificateless authentication protocol is EUF-CMA for adversary $A_{I}$ and adversary $A_{\mathrm{II}}$.

**Lemma 1.**  *Suppose there is an adversary *$A_{I}$* that can win Game I in polynomial time with probability *ε*, where *$q_{u}$* and *$q_{s}$*, respectively, represent the maximum number of create-user query and TCA partial private key query made by *$A_{I}$*. Then, there will be an Algorithm 5 that can solve the ECDLP problem with the advantage of *${Adv}^{EUF-CMA}\geq {\frac{\varepsilon }{q_{u}}(1-\frac{1}{q_{u}})}^{q_{s}}$.

**Proof.**  Suppose $A_{I}$ is an adversary who breaks the unforgeability of the lightweight, certificateless authentication protocol with probability ε in Game I. By constructing Algorithm 5, $A_{I}$ is used as a subroutine, aiming to solve ECDLP. That is, challenger C obtains an ECDLP instance (G,P,Q=sP) and tries to calculate s with the help of $A_{I}$. After the game starts, C maintains lists $L_{u},L_{1},L_{2}$, and $L_{3}$ to track queries to users and oracles. Each list is initially empty. □

Initialization Phase. C randomly selects an identity ${ID}_{g}$ as the target identity, runs the system initialization algorithm to generate public parameter $pp=\{G_{0},G_{1},P,e,p,H_{1},H_{2},H_{3}\}$ and master key $PK=\{Q_{0},Q_{1},Q_{2}\}$, then sends them to the adversary $A_{I}$.

Query Phase. $A_{I}$ performs queries of the following polynomial bounded number.

- Create-user query: For the query with identity ID, C queries the list $L_{u}$. If there is a corresponding entry in the list, ${UPK}_{ID}$ is returned. Otherwise, if $ID\neq {ID}_{g}$, C generates a pseudonym PID, randomly selects $d_{ID},h_{1},x_{ID}\in Z_{p}^{*}$, calculates $R_{ID}=d_{ID}P-Q_{2}h_{1},X_{ID}=x_{ID}P$, and sets $h_{1}=H_{1}(PID|\left|R_{ID}\right||Q_{2})$. If $ID={ID}_{g}$, C randomly selects $r_{ID},x_{ID},h_{1}\in Z_{p}^{*}$, and computes $R_{ID}=r_{ID}P,X_{ID}=x_{ID}P,h_{1}=H_{1}(PID|\left|R_{ID}\right||Q_{2})$. Finally, C sends ${UPK}_{ID}=(R_{ID},X_{ID})$ and pseudonym PID to $A_{I}$, and adds $\left(ID,PID,R_{ID},X_{ID},d_{ID},x_{ID},h_{1}\right)$ to the list $L_{u}$.
- $H_{1}$ query: When receiving the query $H_{1}\left(PID|\left|R_{ID}\right||Q_{2}\right)$ from $A_{I}$, C queries the list $L_{1}$. If $\left(PID,R_{ID},Q_{2},h_{1}\right)$ is in the list, it returns $h_{1}$ to $A_{I}$. Otherwise, C performs the create-user operation and extracts $h_{1}$ from $L_{u}$ and returns it to $A_{I}$.
- $H_{2}$ query: When receiving the query $H_{2}(m|\left|T\right||PID|\left|Q_{2}\right||R_{ID}||X_{ID})$ from $A_{I}$, C queries the list $L_{2}$. If the query value $\left(m,T,PID,Q_{2},R_{ID},X_{ID},h_{2}\right)$ is in list, then C returns $h_{2}$ to $A_{I}$. Otherwise, C randomly selects $h_{2}\in Z_{p}^{*}$ and sets $h_{2}=H_{2}(m|\left|T\right||PID|\left|Q_{2}\right||R_{ID}||X_{ID})$. Finally, C returns $h_{2}$ to $A_{I}$.
- $H_{3}$ query: When receiving a request $H_{3}(V)$ from $A_{I}$, C queries the list $L_{3}$. If the query value V exists in the list $L_{3}$, it returns $h_{3}$ to $A_{I}$. Otherwise, C selects the random number $h_{3}\in Z_{p}^{*}$, sets $h_{3}=H_{3}(V)$, and returns $h_{3}$ to $A_{I}$.
- TCA partial private key query: When receiving the query $\left(PID,d_{ID}\right)$ from $A_{I}$, if $PID=P{ID}_{g}$, C returns ⊥ and terminates the game. Otherwise, C searches list $L_{u}$ and returns $d_{ID}$ to $A_{I}$.
- User private key generation query: When receiving a request $\left(PID,x_{ID}\right)$ from $A_{I}$, C queries the list $L_{u}$. If $\left(PID,x_{ID}\right)$ is in the list, then it returns $x_{ID}$ to $A_{I}$. Here, when the user’s public key is replaced and $A_{I}$ does not provide the corresponding $x_{ID}$, the command to extract the user’s private key does not output the user’s private key.
- User public key replacement query: When receiving the query $\left(PID,R_{ID}^{\prime },X_{ID}^{\prime }\right)$ from $A_{I}$, C queries the list $L_{u}$ and replaces the existing public key $\left(R_{ID},X_{ID}\right)$ with $\left(R_{ID}^{\prime },X_{ID}^{\prime }\right)$, where $x_{ID}$ is set to ⊥.
- Message authentication encryption query: C checks whether these three tuples, $\left(PID,R_{ID},X_{ID},d_{ID},x_{ID},h_{1}\right)$, $\left(PID,R_{ID},Q_{2},h_{1}\right),$ and $\left(m,T,PID,Q_{2},R_{ID},X_{ID},h_{2}\right)$, are in the lists $L_{u},L_{1}$, and $L_{2}$, respectively.

If $PID\neq {PID}_{g}$ and $x_{ID}\neq ⊥$, C randomly selects $k,h_{1},h_{2}\in Z_{p}^{*}$, and sets $h_{1}=H_{1}\left(PID,R_{ID},Q_{2}\right),h_{2}=H_{2}\left(m\left|\left|T\right|\right|PID\left|\left|Q_{2}\right|\right|R_{ID}||X_{ID}\right)$, then calculates T=kP and $\delta =\frac{x_{ID}+k+h_{2}d_{ID}}{x_{ID}}$.

If $PID=P{ID}_{g}$ or $x_{ID}=⊥$, C randomly selects $\delta ,h_{1},h_{2}\in Z_{p}^{*}$, computes $T=\delta X_{ID}-X_{ID}-h_{2}(R_{ID}+h_{1}Q_{2})$, and outputs Cipher1=(T,δ). Then, C adds $\left(PID,R_{ID},X_{ID},d_{ID},x_{ID},h_{1}\right)$, $\left(PID,R_{ID},Q_{2},h_{1}\right)$, and $\left(m,T,PID,Q_{2},R_{ID},X_{ID},h_{2}\right)$ to the lists $L_{u},L_{1}$, and $L_{2}$, respectively.

Forgery Phase. $A_{I}$ provides a valid message ciphertext tuple ${Cipher1}^{\left(1\right)}=(T^{*},\delta^{\left(1\right)}))$ for ${PID}^{*}$. This tuple can be replaced by $A_{I}$; that is, the equation $\delta^{\left(1\right)}X_{ID}^{*}=X_{ID}^{*}+T^{*}+h_{2}^{\left(1\right)}(R_{ID}^{*}+h_{1}^{*}Q_{2})$. At the same time, $A_{I}$ does not allow the TCA partial private key query and message authentication encryption query. If $PID={PID}_{g}$, C terminates the game. According to the forking lemma, we can obtain the other two valid ciphertexts of the message ${Cipher1}^{\left(2\right)}=(T^{*},\delta^{\left(2\right)})$ and ${Cipher1}^{\left(3\right)}=(T^{*},\delta^{\left(3\right)})$, which satisfies:

(5) $$\delta^{\left(2\right)}X_{ID}^{*}=X_{ID}^{*}+T^{*}+h_{2}^{\left(2\right)}\left(R_{ID}^{*}+h_{1}^{*}Q_{2}\right)$$

(6) $$\delta^{\left(3\right)}X_{ID}^{*}=X_{ID}^{*}+T^{*}+h_{2}^{\left(3\right)}\left(R_{ID}^{*}+h_{1}^{*}Q_{2}\right)$$

For convenience, we set $\rho =\left(\delta^{\left(1\right)}-\delta^{\left(2\right)}\right)\left(h_{2}^{\left(1\right)}-h_{2}^{\left(3\right)}\right)-(\delta^{\left(1\right)}-\delta^{\left(3\right)})(h_{2}^{\left(1\right)}-h_{2}^{\left(2\right)})$, and from Equations (5) and (6), we can obtain:

(7) $$\left(\delta^{\left(1\right)}-\delta^{\left(2\right)}\right)x_{ID}^{*}=\left(h_{2}^{\left(1\right)}-h_{2}^{\left(2\right)}\right)\left(r_{ID}^{*}+h_{1}^{*}s\right)$$

(8) $$\left(\delta^{\left(1\right)}-\delta^{\left(3\right)}\right)x_{ID}^{*}=\left(h_{2}^{\left(1\right)}-h_{2}^{\left(3\right)}\right)\left(r_{ID}^{*}+h_{1}^{*}s\right)$$

Therefore,

(9) $$x_{ID}^{*}=\frac{h_{2}^{\left(1\right)}-h_{2}^{\left(2\right)}}{\delta^{\left(1\right)}-\delta^{\left(2\right)}}\left(r_{ID}^{*}+h_{1}^{*}s\right)$$

Taking $x_{ID}^{*}$ into Equation (10), we can obtain:

(10) $$\rho \left(r_{ID}^{*}+h_{1}^{*}s\right)=0,s=-\frac{r_{ID}^{*}}{h_{1}^{*}}$$

This is a solution for the ECDLP instance. Below, we discuss the probability of C winning in Game I. C will win when the following three events occur simultaneously:

E1: When $A_{I}$ queries the TCA partial private key, C does not terminate the game.

E2: ${Cipher1}^{*}$ is a valid forged message ciphertext.

E3: For the forged message ciphertext $\left(T^{*},\delta^{*}\right)$ submitted by $A_{I}$ in the forgery stage, there is $P{ID}^{*}={PID}_{g}$.

We can obtain $\mathrm{Pr}\left[E1\right]\geq {\left(1-\frac{1}{q_{u}}\right)}^{q_{s}}$, $\mathrm{Pr}\left[E2|E1\right]\geq \varepsilon$, and $\mathrm{Pr}\left[E3|E1E2\right]\geq \frac{1}{q_{u}}$. Therefore, the advantage of C is:

(11) $$\begin{array}{ll} {Adv}^{EUF-CMA} & \geq \mathrm{Pr}\left[E1E2E3\right]=\mathrm{Pr}\left[E1\right]\mathrm{Pr}\left[E2|E1\right]\mathrm{Pr}\left[E3|E1E2\right]\\ & \geq {\frac{\varepsilon }{q_{u}}(1-\frac{1}{q_{u}})}^{q_{s}} \end{array}$$

**Lemma 2.**  *Suppose there is an adversary *$A_{\mathrm{II}}$* that can win Game II in polynomial time with probability *ε*, where *$q_{u},q_{s}$*, and *$q_{r}$*, respectively, represent the maximum number of create-user query, user private key generation query, and user public key replacement query made by *$A_{\mathrm{II}}$*. Then, there will be an Algorithm 6 that can solve the ECDLP problem with the advantage of *${Adv}^{EUF-CMA}\geq {\frac{\varepsilon }{q_{u}}(1-\frac{1}{q_{u}})}^{q_{s}+q_{r}}$.

**Proof.**  Suppose $A_{\mathrm{II}}$ is an adversary who breaks the unforgeability of the lightweight, certificateless authentication protocol with probability ε in Game II. By constructing Algorithm 6, $A_{\mathrm{II}}$ is used as a subroutine, aiming to solve ECDLP. That is, challenger C obtains an ECDLP instance ($G,P,Q=x_{ID}P$) and tries to calculate $x_{ID}$ with the help of $A_{\mathrm{II}}$. After the game starts, C maintains lists $L_{u},L_{1},L_{2}$, and $L_{3}$ to track queries to users and oracles. Each list is initially empty. □

Initialization Phase. C randomly selects an identity ${ID}_{g}$ as the target identity and runs the system initialization algorithm to generate public parameter $pp=\{G_{0},G_{1},P,e,p,H_{1},H_{2},H_{3}\}$. Then, C selects $s\in Z_{p}^{*}$, sets $Q_{2}=sP$, generates master key $PK=\{Q_{0},Q_{1},Q_{2}\}$, and sends (pp,PK,s) to the adversary $A_{\mathrm{II}}$.

Query Phase. $A_{\mathrm{II}}$ performs queries of the following polynomial bounded number.

- Create-user query: For the query with identity ID, C queries the list $L_{u}$. If there is a corresponding entry in the list, ${UPK}_{ID}$ is returned. Otherwise, C generates a pseudonym PID, randomly chooses $r_{ID},h_{1}\in Z_{p}^{*}$, computes $R_{ID}=r_{ID}P$ and $d_{ID}=r_{ID}+h_{1}s$, then sets $h_{1}=H_{1}(PID|\left|R_{ID}\right||Q_{2})$. If $ID\neq {ID}_{g}$, C randomly selects $x_{ID}\in Z_{p}^{*}$ and calculates $X_{ID}=x_{ID}P$. If $ID={ID}_{g}$, C sets $X_{ID}=x_{ID}P$ and $x_{ID}=⊥$. Finally, C sends ${UPK}_{ID}=(R_{ID},X_{ID})$ and pseudonym PID to $A_{\mathrm{II}}$, and adds $\left(ID,PID,R_{ID},X_{ID},d_{ID},x_{ID},h_{1}\right)$ to the list $L_{u}$.
- $H_{1}$ query: When receiving the query $H_{1}\left(PID|\left|R_{ID}\right||Q_{2}\right)$ from $A_{\mathrm{II}}$, C queries the list $L_{1}$. If $\left(PID,R_{ID},Q_{2},h_{1}\right)$ is in the list, it returns $h_{1}$ to $A_{\mathrm{II}}$. Otherwise, C performs the create-user operation and extracts $h_{1}$ from $L_{u}$ and returns it to $A_{\mathrm{II}}$.
- $H_{2}$ query: When receiving the query $H_{2}(m|\left|T||PID||Q_{2}||R_{ID}\right||X_{ID})$ from $A_{\mathrm{II}}$, C queries the list $L_{2}$. If the query value $\left(m,T,PID,Q_{2},R_{ID},X_{ID},h_{2}\right)$ is in the list, then C returns $h_{2}$ to $A_{\mathrm{II}}$. Otherwise, C randomly selects $h_{2}\in Z_{p}^{*}$ and sets $h_{2}=H_{2}(m|\left|T||PID||Q_{2}||R_{ID}\right||X_{ID})$. Finally, C returns $h_{2}$ to $A_{\mathrm{II}}$.
- $H_{3}$ query: When receiving a request $H_{3}(V)$ from $A_{\mathrm{II}}$, C queries the list $L_{3}$. If the query value V  exists in the list $L_{3}$, it returns $h_{3}$ to $A_{\mathrm{II}}$. Otherwise, C selects the random number $h_{3}\in Z_{p}^{*}$, sets $h_{3}=H_{3}(V)$, and returns $h_{3}$ to $A_{\mathrm{II}}$.
- TCA partial private key query: When receiving the query $\left(PID,d_{ID}\right)$ from $A_{\mathrm{II}}$, C searches list $L_{u}$, and if the entry exists, C returns $d_{ID}$ to $A_{\mathrm{II}}$. Otherwise, C performs a create-user query using the identification PID and returns $d_{ID}$ to $A_{\mathrm{II}}$.
- User private key generation query: When receiving a request $\left(PID,x_{ID}\right)$ from $A_{\mathrm{II}}$, if $PID=P{ID}_{g}$, C returns ⊥ and terminates the game. Otherwise, C searches list $L_{u}$ and returns $x_{ID}$ to $A_{\mathrm{II}}$.
- User public key replacement query: When receiving the query $\left(PID,R_{ID}^{\prime },X_{ID}^{\prime }\right)$ from $A_{I}$, if $PID=P{ID}_{g}$, C returns ⊥ and terminates the game. Otherwise, C updates the list $\left(PID,R_{ID},X_{ID},d_{ID},x_{ID},h_{1}\right)$ to $\left(PID,R_{ID}^{\prime },X_{ID}^{\prime },d_{ID},x_{ID},h_{1}\right)$, where $x_{ID}$ is set to ⊥.
- Message authentication encryption query: C checks whether these three tuples, $\left(PID,R_{ID},X_{ID},d_{ID},x_{ID},h_{1}\right)$, $\left(PID,R_{ID},Q_{2},h_{1}\right)$, and $\left(m,T,PID,Q_{2},R_{ID},X_{ID},h_{2}\right)$, are in the lists $L_{u},L_{1}$, and $L_{2}$, respectively.

If $PID\neq {PID}_{g}$ and $x_{ID}\neq ⊥$, C randomly selects $k,h_{1},h_{2}\in Z_{p}^{*}$, and sets $h_{1}=H_{1}\left(PID||R_{ID}||Q_{2}\right),h_{2}=H_{2}\left(m|\left|T\right||PID|\left|Q_{2}\right||R_{ID}||X_{ID}\right)$, then calculates T=kP and $\delta =\frac{x_{ID}+k+h_{2}d_{ID}}{x_{ID}}$.

If $PID={PID}_{g}$ or $x_{ID}=⊥$, C randomly selects $\delta ,h_{1},h_{2}\in Z_{p}^{*}$, computes $T=\delta X_{ID}-X_{ID}-h_{2}(R_{ID}+h_{1}Q_{2})$, and outputs Cipher1=(T,δ). Then, C adds $\left(PID,R_{ID},X_{ID},d_{ID},x_{ID},h_{1}\right)$, $\left(PID,R_{ID},Q_{2},h_{1}\right)$, and $\left(m,T,PID,Q_{2},R_{ID},X_{ID},h_{2}\right)$ to the lists $L_{u},L_{1}$, and $L_{2}$, respectively.

Forgery Phase. $A_{\mathrm{II}}$ provides a valid message ciphertext tuple, ${Cipher1}^{\left(1\right)}=(T^{*},\delta^{\left(1\right)}))$, for $P{ID}^{*}$. At the same time, $A_{\mathrm{II}}$ has never used $P{ID}^{*}$ for the user private key generation query and user public key replacement query and has never used ${(M^{*},PID}^{*})$ for the message authentication encryption query. If $PID\neq P{ID}_{g}$, C terminates the game. Otherwise, C will search for the tuples $\left({PID}^{*},R_{ID}^{*},X_{ID}^{*},d_{ID}^{*},x_{ID}^{*},h_{1}^{*}\right)$, $\left(P{ID}^{*},R_{ID}^{*},Q_{2},h_{1}^{*}\right)$, and $\left(m^{*},T^{*},P{ID}^{*},Q_{2},R_{ID}^{*},X_{ID}^{*},h_{2}^{*}\right)$ in the lists $L_{u},L_{1}$, and $L_{2}$, respectively. According to the forking lemma, we can obtain the other two valid ciphertexts of the message ${Cipher1}^{\left(2\right)}=(T^{*},\delta^{\left(2\right)})$, which satisfies:

(12) $$\delta^{\left(2\right)}X_{ID}^{*}=X_{ID}^{*}+T^{*}+h_{2}^{\left(2\right)}\left(R_{ID}^{*}+h_{1}^{*}Q_{2}\right)\;$$

Below, we discuss the probability of C winning in Game II. C will win when the following three events occur simultaneously:

E1: When $A_{\mathrm{II}}$ performs user private key generation query and user public key replacement query, C does not terminate the game.

E2: ${Cipher1}^{*}$ is a valid forged message ciphertext.

E3: For the forged message ciphertext $\left(T^{*},\delta^{*}\right)$ submitted by $A_{\mathrm{II}}$ in the forgery stage, there is ${PID}^{*}={PID}_{g}$.

It is easy to obtain $\mathrm{Pr}\left[E1\right]\geq {\left(1-\frac{1}{q_{u}}\right)}^{q_{s}}{\left(1-\frac{1}{q_{u}}\right)}^{q_{r}}$, $\mathrm{Pr}\left[E2|E1\right]\geq \varepsilon$, and $\mathrm{Pr}\left[E3|E1E2\right]\geq \frac{1}{q_{u}}$. Therefore, the advantage of C is:

(13) $$\begin{array}{ll} {Adv}^{EUF-CMA} & \geq \mathrm{Pr}\left[E1E2E3\right]=\mathrm{Pr}\left[E1\right]\mathrm{Pr}\left[E2|E1\right]\mathrm{Pr}\left[E3|E1E2\right]\\ & \geq {\frac{\varepsilon }{q_{u}}(1-\frac{1}{q_{u}})}^{q_{s}+q_{r}} \end{array}$$

#### 5.1.3. Informal Security Analysis

(1) Bidirectional identity authentication

Our authentication protocol can achieve bidirectional identity authentication between Data_Sender and Data_Receiver. Data_Sender uses Data_Receiver’s public key to encrypt the message. Only Data_Receiver’s private key can successfully decrypt the message ciphertext, thus verifying the identity of Data_Receiver. Meanwhile, Data_Receiver uses the public key of the Data_Sender to verify the identity of the message sender. If the formula $\delta X_{S}=X_{S}+T+h_{2}(R_{S}+h_{1}^{\prime }Q_{2})$ is workable, the identity of the Data_Sender can be confirmed. Therefore, the authentication protocol realizes the mutual identity authentication.

(2) Anonymity

In the authentication protocol, the real identity of the user is ${ID}_{i}$. During the user registration phase, TCA randomly generates a pseudonym $P{ID}_{i}$ for user ${ID}_{i}$. The user’s true identity is known only to himself and TCA. For any attacker, $P{ID}_{i}$ and ${ID}_{i}$ have no connection, so the corresponding ${ID}_{i}$ cannot be calculated according to $P{ID}_{i}$. In this way, users’ anonymity can be better guaranteed.

(3) Traceability

When there is an abnormality in the identity of ${PID}_{i}$, that is, there are some dangerous behaviors, it is necessary to obtain the true identity of the user. The investigator can obtain the true identity of the user by sending ${PID}_{i}$ to TCA. Thus, the protocol guarantees user traceability.

(4) Resist replay attacks

During the authentication phase of the protocol, the random number method is used to resist replay attacks. In each session, all messages sent by the Data_Sender through the public channel contain random numbers k. The random number is used to ensure the freshness and independence of the message ciphertext. The Data_Receiver can detect whether a malicious attacker has launched a message replay attack by checking the random numbers in the received message. Therefore, our proposed authentication protocol is resistant to replay attacks.

(5) Resist Man-in-the-Middle attacks

During the identity authentication process between TCA and DU, the attacker disguises himself as a legitimate communicator and attempts to communicate with TCA or DU. The message ciphertext transmitted during the authentication process is Cipher=(T,C,δ). If the attacker wants to pretend to be a legitimate user, he needs to know the initial values of the variables $k,x_{S}$ and $d_{S}$. k is a random number generated by Data_Sender, which cannot be accomplished by random guessing alone. Calculating the value of k based on the intercepted T is a discrete logarithm problem, so the attacker cannot find k in polynomial time. In addition, $x_{S}$ and $d_{S}$ are private keys of Data_Sender, which are saved locally and attackers cannot obtain them. Therefore, an attacker cannot disguise himself as a legitimate user, that is, the authentication protocol resists man-in-the-middle attacks.

### 5.2. Data Security Analysis

(1) Data confidentiality

Data in the SDACS will be encrypted and uploaded to the IPFS. The characteristics of IPFS distributed storage and content-based addressing can achieve permanent data storage and data tamper-proofing. Besides, only the DU who meets the access policy requirements set by the DO can obtain the corresponding ciphertext address.

In the blockchain, users inevitably need to provide identity data information for data sharing. Although users can selectively provide identity information to the blockchain, they still face the problem of how to minimize the disclosure of information to achieve privacy protection. Therefore, we separate on-chain credentials and off-chain credentials so that only the TCA and the user themselves have real identity data to avoid exposing privacy on the chain. When the DU obtains the ciphertext from the IPFS, it sends a decryption key request to the TCA. The two parties send information through a lightweight identity authentication protocol, which verifies the identity and access rights while ensuring the security of off-chain information transmission. Since the decryption key and ciphertext are obtained separately, malicious attackers need to attack the two interactive processes at the same time to obtain data, which greatly increases the difficulty of the attack and ensures the confidentiality of the data.

(2) Data immutability

The DO stores the data hash and storage address of the ciphertext into the blockchain together. Since the ledger data of the blockchain are immutable, that is, the stored hash value cannot be tampered with, once the data resource is changed, the TCA can determine whether the DU has the authority to obtain the decryption key by verifying hashdata.

(3) Data security

For the given ciphertext $CT=\{C_{m}=m\cdot e(P,P{)}^{\alpha \theta },C_{1}=-\theta \beta P,C_{2}=\theta P\}$, the public parameter $pp=\{G_{0},G_{1},P,e,p,H_{1},H_{2},H_{3}\}$, and master key $PK=\{Q_{0}=e(P,P{)}^{\alpha },Q_{1}=\beta P,Q_{2}=sP\}$, if the attacker wants to crack the ciphertext $C_{m}$, the value of $e(P,P{)}^{\alpha \theta }$ needs to be calculated. The attacker can obtain $e(P,P{)}^{\alpha }$ from the master key. Nevertheless, the parameter θ is a private parameter selected and saved by the DO. In addition, the process by which the attacker calculates the parameter θ based on θP and the generator P is a discrete logarithm problem, so it is impossible to calculate θ based on $C_{2}$ in arbitrary polynomial time. Therefore, the attacker cannot obtain the corresponding data based on the given ciphertext, public parameters, and master key, which ensures the data security.

## 6. Performance Evaluation

In this section, we implement a prototype to evaluate the feasibility and effectiveness of the SDACS. All our programs are executed on VMware @Workstation Pro 16.1.2 with a dual-core Intel Core i5-7500CPU@3.40 GHz and 8.0 GB RAM running Ubuntu 20.04. In the off-chain part, we compare the computational and communication overhead of the authentication protocol, as well as encryption and decryption processes to verify the effectiveness of the SDACS. In the on-chain part, we evaluate SDACS based on the functional implementation, throughput, and transaction latency of smart contracts.

### 6.1. Authentication Evaluation

We compared the proposed lightweight certificateless authentication protocol with other certificateless authentication schemes for IoT. We evaluated the performance of the authentication scheme by calculating the computational and storage overhead in different schemes, including verifying message encryption and decryption times and the message ciphertext size. When evaluating the authentication protocol, the curve was chosen as the super singular elliptic curve, $E:y^{2}=x^{3}+x\;mod\;p_{1}$, on a finite field $F_{p}$ with p of 512-bit prime numbers. The curve parameters used in our protocol are shown in Table 4. Additionally, we used the Python-based Pairing-Based Cryptography (PBC) library to calculate the running time of encryption and decryption operations. We indicate the running time of relevant operations in Table 5. The comparison scheme is the certificateless authentication protocol of Basudan et al. [29], Barbosa et al. [30], Eslami et al. [31], and Liao et al. [32].

#### 6.1.1. Computational Cost

**Figure 4:**
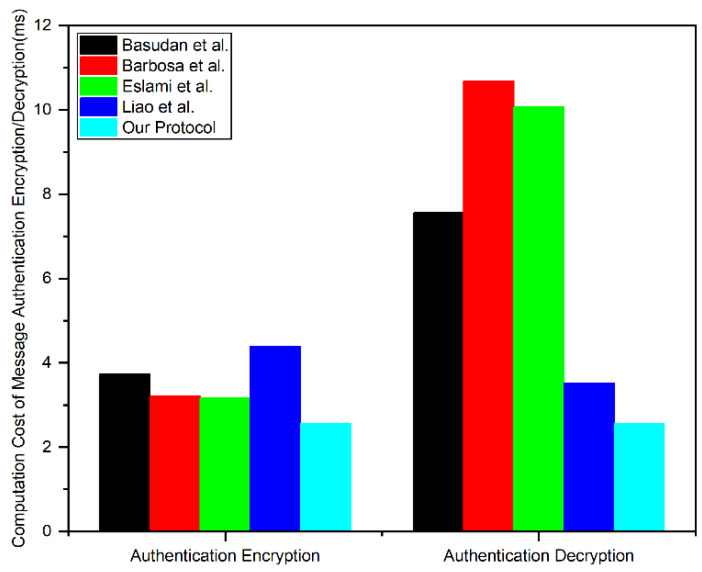
Computational cost of message authentication encryption/decryption [29,30,31,32].

We evaluated the computational cost of these protocols by calculating the running time of the message authentication encryption and message authentication decryption stages. Table 6 shows the computational cost for different protocols. We can see that our authentication protocol has lower costs compared to all protocols because we aimed to reduce consumption between the DU and TCA as much as possible. In the message authentication encryption and decryption phases, the message sender needs to perform four scalar multiplication operations on the elliptic curve and three hash operations mapped to $Z_{p}^{*}$ each time. The computational cost comparison of message authentication encryption/decryption of the protocol is shown in Figure 4. Evidence shows that our protocol performed well in both the cost of message authentication encryption and decryption stages. In the message authentication decryption stage, our protocol had the lowest computational cost, which is suitable for scenarios with limited device computing capabilities.

#### 6.1.2. Communication Cost

In the authentication protocol, the message sender runs the message authentication encryption algorithm to encrypt the sent information and sends the message ciphertext to the receiver. The message receiver runs the message authentication decryption algorithm to confirm the identity of the message sender and decrypt it. We evaluated the communication costs of the protocols by comparing the sizes of the message ciphertext. The communication cost comparison of different protocols is shown in Table 7. We can see that the communication overhead of our proposed protocol was relatively small. Liao et al.’s [32] protocol maps messages into $Z_{p}^{*}$, and the message receiver cannot decrypt the message itself. It only implements the identity authentication function and ignores the transmission of new data.

#### 6.1.3. Storage Cost

In order to store key data used for authentication, the storage cost of the protocol is a significant part of the mutual authentication between the user and TCA. We compare the storage cost of the authentication protocol with other related schemes, as shown in Table 8. In our protocol, the user needs to store his pseudonym $P{ID}_{i}$ and real identity ${ID}_{i}$ locally. Assuming that the lengths of $P{ID}_{i}$ and ${ID}_{i}$ are x bits. In addition, the user also needs to store the public key ${UPK}_{i}=(R_{i},X_{i})$ and private key ${UPK}_{i}=(R_{i},X_{i})$, where ${UPK}_{i}\in G_{0}$ and ${USK}_{i}\in Z_{p}^{*}$. Therefore, the user’s storage overhead is $2\left|G_{0}\right|+2\left|Z_{p}^{*}\right|+2x=2368+2x\;bits$. Although the storage overhead of our authentication protocol is relatively large, our protocol assigns a pseudonym to each user, achieving anonymity of user identity, which is not applied by other protocols.

According to the above results, our lightweight, certificateless authentication protocol performed better than existing protocols in terms of computational cost, communication cost and storage cost.

### 6.2. Encryption and Decryption Evaluation

In the data encryption and decryption process, the DO stores the access policy in the blockchain, and users only need to perform a few operations. The access policy matching process is automatically executed by the smart contracts, thus reducing the user’s computing overhead. We experimentally tested the time of data encryption, decryption key generation, and data decryption. To show the advantage of our solution, we compared it with Li et al.’s scheme [33]. The experimental configuration is the same as in Section 6.1. The value measured in the experiment is the average time consumed by executing each type of operation 50 times.

**Figure 5:**
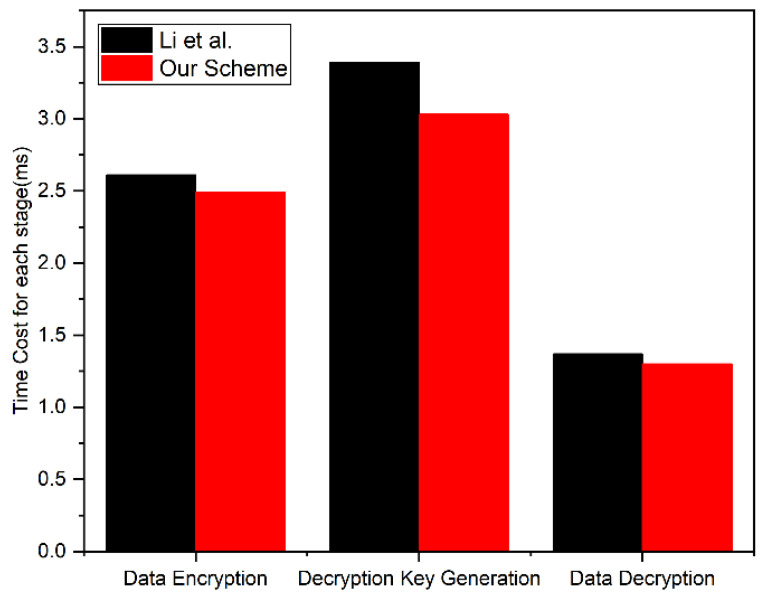
Time cost in data encryption, decryption key generation, and data decryption [33].

As shown in Figure 5, our scheme was more efficient than Li et al.’s [33]. The ciphertext of both schemes does not contain the access policy. However, in Li et al.’s [33] scheme, the number of parameters contained in the decryption key increases as the number of access policies increases. When the access policies continue to increase, the decryption key generation time and data decryption time increase accordingly, which will undoubtedly bring greater computational overhead. In the above experiments, we set the number of the access policy to 1, which is the minimum time consumption of Li et al.’s [33] scheme. Our scheme ensures both efficiency and safety. On the one hand, we performed bilinear pairing based on the additive cyclic group. Compared with the multiplicative cyclic group used by Li et al. [33], the operation was faster, thus reducing the computational overhead. On the other hand, the decryption key generation requires the input of the TCA’s master key (which the DU cannot obtain), so the DU cannot generate the decryption key by itself, thus ensuring the security of data decryption.

### 6.3. Access Control Evaluation

To test the performance of the distributed dynamic access control scheme, we built a blockchain test platform with 24 peer nodes by using Hyperledger Fabric v1.4. The hardware and software environments of the experiment are shown in Table 9.

#### 6.3.1. System and Function Display

**Figure 6:**
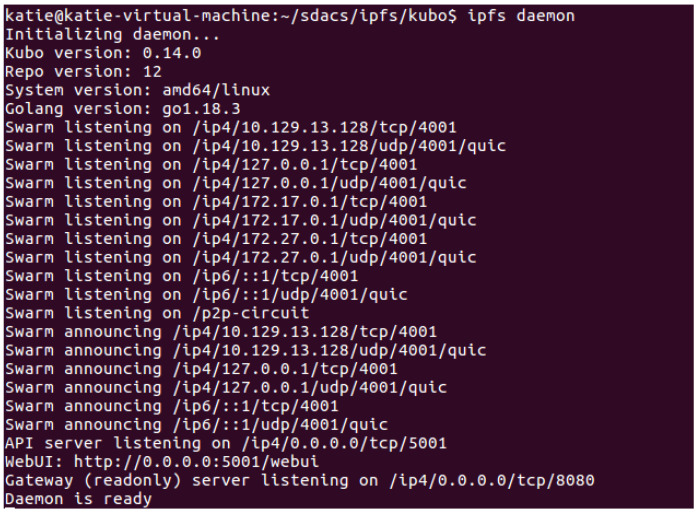
IPFS startup process.

Users can upload and download data according to the operating mechanism of the IPFS. After the DO adds the IPFS v0.14.0 installation directory to the environment variable, it can enter “ipfs daemon” in the terminal to start the IPFS, as shown in Figure 6.

**Figure 7:**
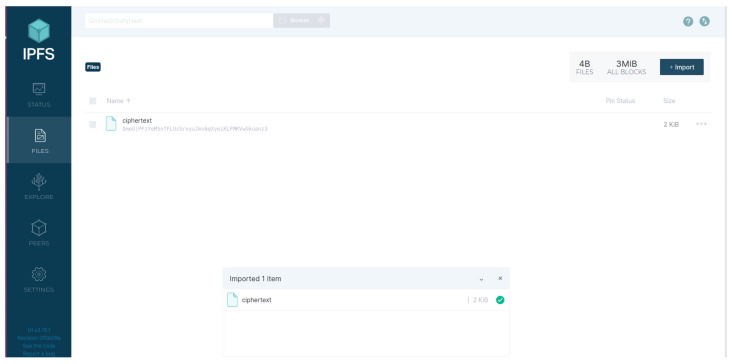
Data upload interface in the IPFS.

Users upload and download data resources through IPFS WebUI. As shown in Figure 7, the DO stores ciphertext in the IPFS and obtains the content identifier (CID): QmeGjPFzYeM5nfFLUsSrxyuJms6gXyeiKLFMKVwGkuanz3. Then, the DU uploads the CID as a data resource to the blockchain network.

**Figure 8:**
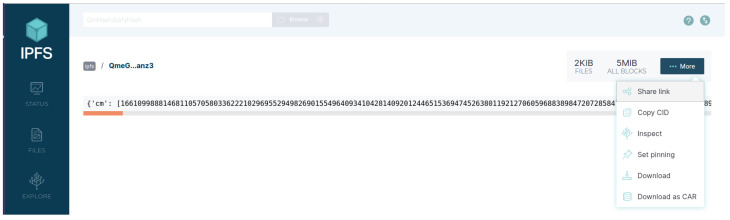
Data download interface in the IPFS.

After the DU obtains the ciphertext address, it can search and download the ciphertext by entering the CID, as shown in Figure 8.

We tested the system functions from three aspects: data resource storage, policy management, and access control.

**Figure 9:**
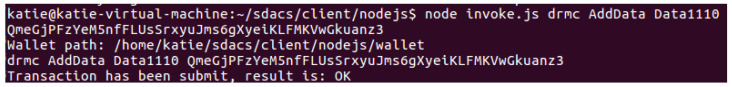
Result of invoking the AddData() method.

Data resource storage test: As shown in Figure 9, the DO invokes the AddData() method in the DRMC to upload the data resource to the blockchain network.

**Figure 10:**
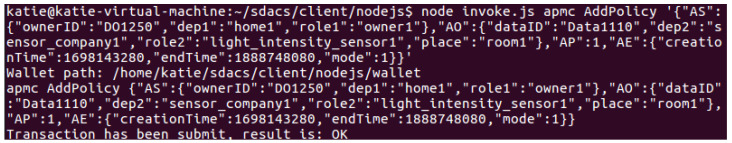
Result of invoking the AddPolicy() method.

**Figure 11:**
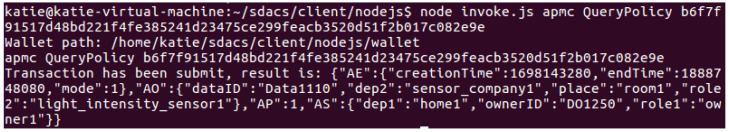
Result of invoking the QueryPolicy() method.

**Figure 12:**
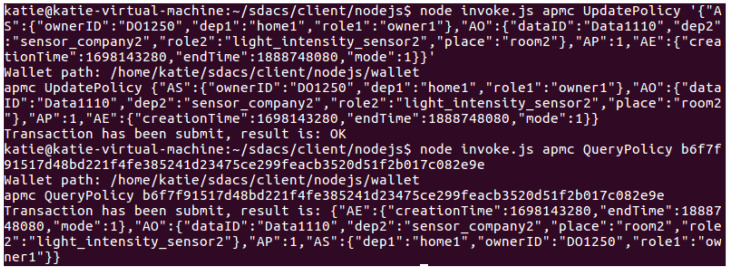
Result of invoking the UpdatePolicy() method.

**Figure 13:**
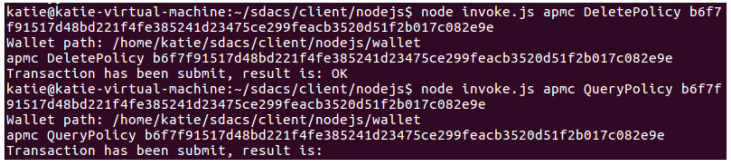
Result of invoking the DeletePolicy() method.

Policy management test: As shown in Figure 10, after uploading the data resource, the DO calls the AddPolicy() method in the APMC to upload the access policy. As depicted in Figure 11, the DO can call the QueryPolicy() method to query the access policy. The key used in the query is ResourceID, which can be obtained according to the following formula: ResourceID=SHA256(OwnerID,DataID). Besides, the DO can call the UpdatePolicy() method to update the policy (reference Figure 12 for more details). Figure 13 shows how the DO deletes the access policy through the DeletePolicy() method.

**Figure 14:**
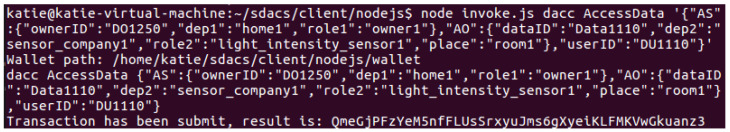
Result of invoking the AccessData() method.

**Figure 15:**
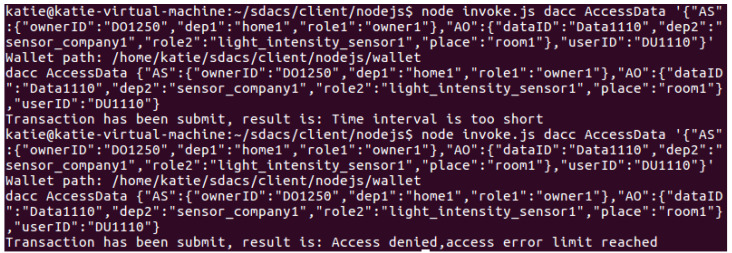
Result of sending frequent access requests.

Access control test: The DU makes an access request to the DO’s data resource and invokes the CheckAccess() method of the DACC to perform permission detection. The DACC checks user attributes from four aspects: misbehavior verification, time verification, permission verification, and attribute matching. If the detection passes, the data resource will be returned to the DU, as shown in Figure 14. Figure 15 shows the result of the DU being punished as a malicious visitor by the blockchain network for sending frequent access requests.

#### 6.3.2. Performance Testing

**Figure 16:**
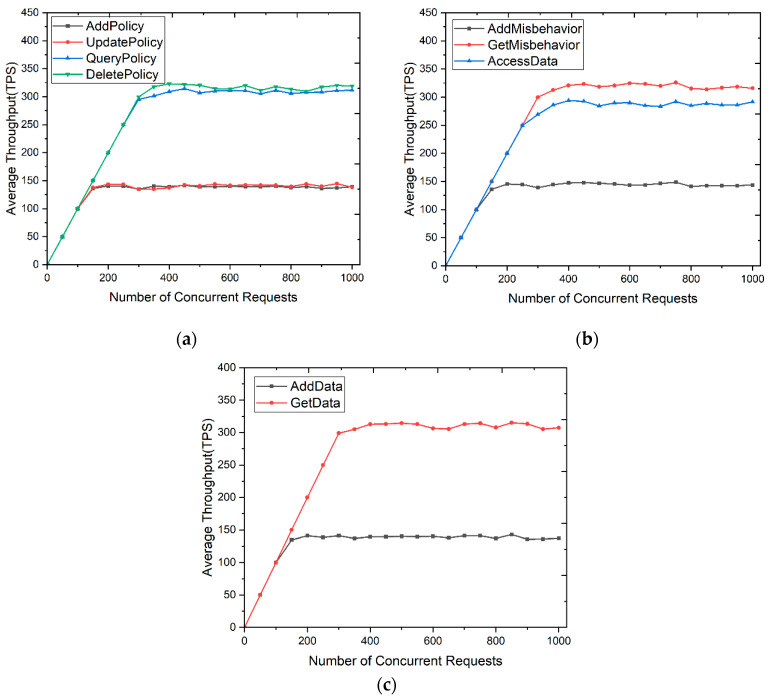
(**a**) Average throughput performance of the APMC with different concurrent requests. (**b**) Average throughput performance of the DACC with different concurrent requests. (**c**) Average throughput performance of the DRMC with different concurrent requests.

To test the performance of the SDACS, we designed two sets of experiments to demonstrate the impact of the number of concurrent requests by multi-threaded clients on the system’s smart contracts’ throughput and transaction latency. The experiments set that all simulated user requests have data access and read permissions, and the access policies and requests in each access control scheme were the same. The range of concurrent requests was from 0 to 1000. Then, the average throughputs of smart contracts are disclosed in Figure 16.

Read operations have higher throughput than write operations. For write operations, after the data location is found based on the data index value, a historical version of the old data needs to be generated for strict verification and accurate tracing. As can be seen from Figure 16a, the throughput of DeletePolicy() and QueryPolicy() in the APMC was higher than that of AddPolicy() and UpdatePolicy(). Figure 16b illustrates that the throughput of GetMisbehavior() and AccessData() in the DACC was higher than that of AddMisbehavior(). As shown in Figure 16c, the throughput of GetData() in the DRMC was higher than AddData().

When the number of concurrent requests was less than 300, the average throughput increased as the number of concurrent requests increased. Upon reaching approximately 300 concurrent requests, the system’s throughput stabilized. This is because the resource utilization of the blockchain reached saturation, but the transactions of a single node will not be affected.

Figure 16b shows that the throughput of AccessData() in the DACC was lower than other smart contracts that focus on read operations. The data access control contract of the SDACS system calls smart contracts named APDC, DRMC, and APMC, meaning that calling another smart contract in a smart contract will reduce the system performance and the overall throughput of the network.

**Figure 17:**
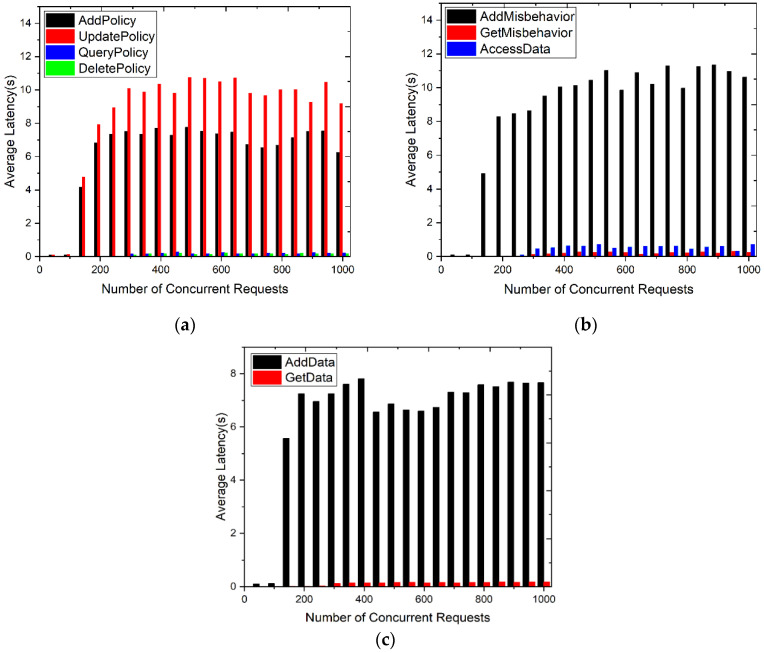
(**a**) Average latency performance of the APMC with different concurrent requests. (**b**) Average latency performance of the DACC with different concurrent requests. (**c**) Average latency performance of the DRMC with different concurrent requests.

Figure 17 shows how the average transaction latency of the system changed with the number of concurrent requests. We can see that when the number of concurrent accesses was greater than 200, the average transaction latency of the system had little relationship with the number of concurrent requests. The latency of read operations on access policy, misbehavior, and data resource was smaller than the latency of write operations. As noted from the graphs, the latency range for read operations was between 0 and 0.7 s, and for write operations between 8 and 11 s. Read operations are more reliable than write operations because they do not involve complex operations, such as data copying and consensus confirmation.

## 7. Conclusions and Future Work

In this study, we established a secure, dynamic, fine-grained IoT data storage and sharing scheme by combining off-chain message authentication transmission and on-chain access control permission verification, named SDACS. For malicious visitors, we proposed a punishment mechanism to impose corresponding behavior restrictions on the user through a misbehavior list. To achieve secure large-scale data storage, our scheme encrypts data and stores it in the IPFS. In addition, we proposed a lightweight, certificateless authentication protocol to minimize the disclosure of identity information and ensure the privacy of the off-chain message sharing process.

We used Hyperledger Fabric and Python to build and implement the proposed scheme, ensuring that only authorized data users can access the data. We also conducted extensive experiments to evaluate the performance of SDACS. Experimental results proved that our scheme has obvious advantages in tamper resistance, fast authentication, and minimum risk of privacy leakage. Additionally, it is suitable for scenarios where multi-party data are securely and efficiently shared in the IoT. In the future, we still need to conduct research on program improvement. For example, we can consider introducing zero-knowledge proof technology in the attribute matching process of data users, so that no information about attributes will be revealed during the permission verification process, thereby achieving a higher level of privacy protection.

## Figures and Tables

**Table 1:** Comparisons of major blockchain-based access control schemes.

Reference	Basic Model	Blockchain Platform	Storage Platform	Identity Privacy Protection	Application
[15]	CapBAC, IBAC	Ethereum	-	No	IoT
[16]	ABAC	Ethereum	Blockchain	No	Supply Chain
[17]	CapBAC	Ethereum	Storage Device	No	IoT
[20]	ABAC	Ethereum	Resource Pool	No	Common scenarios
[21]	ABAC	Hyperledger Fabric	Edge Blockchain Manager	No	IoT
[22]	ABAC	Hyperledger Fabric	Blockchain	Yes	Healthcare
[24]	ABAC	Hyperledger Fabric	Local Database	No	IoT
[26]	ABAC	Hyperledger Fabric	IPFS	No	IoT
[27]	DAC	Ethereum	IPFS	No	Food Supply
Ours	ABAC	Hyperledger Fabric	IPFS	Yes	IoT

**Table 2:** An example of the access policy.

AS	AO	AP	AE
OwnerID: DO1250	DataID: Data1110	1	CreationTime: 1698143280
Dep1: home1	Dep2: sensor_company1	0	Endtime: 1798748080
Role1: owner1	Role2: light_intensity_sensor1		Mode:1
	Place: room1		

**Table 3:** An example of a misbehavior list.

UserID	DataID	Timestamp	Error Number	Results
DU1110	Data1110	1701332571	2	Time interval is too short.
DU1111	Data1110	1701332654	10	Access denied, access error limit reached, access denied.

**Table 4:** Description of curve parameters.

Curve	Pairing	Group	$\left|Z_{p}^{*}\right|$	Length of Elements of Group
$E:y^{2}=x^{3}+x\;mod\;p_{1}$	$e{:G}_{0}\times G_{0}\to G_{1}$	$G_{0}\left(P\right)$	$\left|Z_{p}^{*}\right|=160\;\mathrm{bits}$	$\left|G_{0}\right|=2\left|p_{1}\right|=1024\;\mathrm{bits}$

**Table 5:** Description and running time of the cryptographic operation.

Cryptographic Operation	Running Time (ms)
Bilinear pairing operation $T_{bp}$	1.87
Scalar multiplication operation $T_{em}$	0.61
Hash operation $T_{h}$	0.04

**Table 6:** Computational cost comparison of different authentication protocols.

Protocol	Message Authentication Encryption Cost	Message Authentication Decryption Cost
Basudan et al. [29]	$6T_{em}+2T_{h}$	$2T_{h}+4T_{bp}$
Barbosa et al. [30]	$2T_{em}+3T_{h}+T_{bp}$	$3T_{h}+5T_{bp}+2T_{em}$
Eslami et al. [31]	$3T_{h}+5T_{em}$	$3T_{h}+5T_{bp}+T_{em}$
Liao et al. [32]	$4T_{em}+2T_{h}+T_{bp}$	$T_{bp}+T_{em}+T_{h}$
Our Protocol	$4T_{em}+3T_{h}$	$4T_{em}+3T_{h}$

**Table 7:** Communication cost comparison of different authentication protocols.

Protocol	Basudan et al. [29]	Barbosa et al. [30]	Eslami et al. [31]	Liao et al. [32]	Our Protocol
Message Ciphertext Length	$2\left|G_{0}\right|+n=2048+n\;bits$	$2\left|G_{0}\right|+n=2048+n\;bits$	$2\left|G_{0}\right|+n=2048+n\;bits$	$3\left|G_{0}\right|+2\left|Z_{p}^{*}\right|=3392\;bits$	$\left|G_{0}\right|+\left|Z_{p}^{*}\right|+n=1184+n\;bits$

**Table 8:** Storage cost comparison of different authentication protocols.

Protocol	Basudan et al. [29]	Barbosa et al. [30]	Eslami et al. [31]	Liao et al. [32]	Our Protocol
Storage Cost of the User	$2\left|G_{0}\right|+2\left|Z_{p}^{*}\right|+x=2368+x\;bits$	$2\left|G_{0}\right|+\left|Z_{p}^{*}\right|+x=2208+x\;bits$	$2\left|G_{0}\right|+\left|Z_{p}^{*}\right|+x=2208+x\;bits$	$3\left|G_{0}\right|+\left|Z_{p}^{*}\right|+x=3232+x\;bits$	$2\left|G_{0}\right|+2\left|Z_{p}^{*}\right|+2x=2368+2x\;bits$

**Table 9:** Hardware and software environments.

Hardware		Software	
CPU	i5-7500 CPU 3.40 GHz	OS	Ubuntu 16.04.7 LTS
Memory	16.00 GB	docker	v20.10.7
Hard Disk	1 TB	docker-compose	v1.17.0
		node	v16.17.0
		golang	v1.18.5
		git	v2.7.4
		Hyperledger Fabric	v1.4

## Data Availability

Data is contained within the article.
